# Analyzing the surface of functional nanomaterials—how to quantify the total and derivatizable number of functional groups and ligands

**DOI:** 10.1007/s00604-021-04960-5

**Published:** 2021-09-04

**Authors:** Daniel Geißler, Nithiya Nirmalananthan-Budau, Lena Scholtz, Isabella Tavernaro, Ute Resch-Genger

**Affiliations:** Bundesanstalt für Materialforschung und -prüfung (BAM), Division Biophotonics (BAM-1.2), Richard-Willstätter-Str. 11, 12489 Berlin, Germany

**Keywords:** Functional group quantification, Surface ligand, Nanomaterial, Nanoparticle, Bead, Dye-based assay, Optical detection, Electrochemical titration, Instrumental analysis, Nanosafety, Safe-by-design

## Abstract

Functional nanomaterials (NM) of different size, shape, chemical composition, and surface chemistry are of increasing relevance for many key technologies of the twenty-first century. This includes polymer and silica or silica-coated nanoparticles (NP) with covalently bound surface groups, semiconductor quantum dots (QD), metal and metal oxide NP, and lanthanide-based NP with coordinatively or electrostatically bound ligands, as well as surface-coated nanostructures like micellar encapsulated NP. The surface chemistry can significantly affect the physicochemical properties of NM, their charge, their processability and performance, as well as their impact on human health and the environment. Thus, analytical methods for the characterization of NM surface chemistry regarding chemical identification, quantification, and accessibility of functional groups (FG) and surface ligands bearing such FG are of increasing importance for quality control of NM synthesis up to nanosafety. Here, we provide an overview of analytical methods for FG analysis and quantification with special emphasis on bioanalytically relevant FG broadly utilized for the covalent attachment of biomolecules like proteins, peptides, and oligonucleotides and address method- and material-related challenges and limitations. Analytical techniques reviewed include electrochemical titration methods, optical assays, nuclear magnetic resonance and vibrational spectroscopy, as well as X-ray based and thermal analysis methods, covering the last 5–10 years. Criteria for method classification and evaluation include the need for a signal-generating label, provision of either the total or derivatizable number of FG, need for expensive instrumentation, and suitability for process and production control during NM synthesis and functionalization.

**Graphical abstract:**

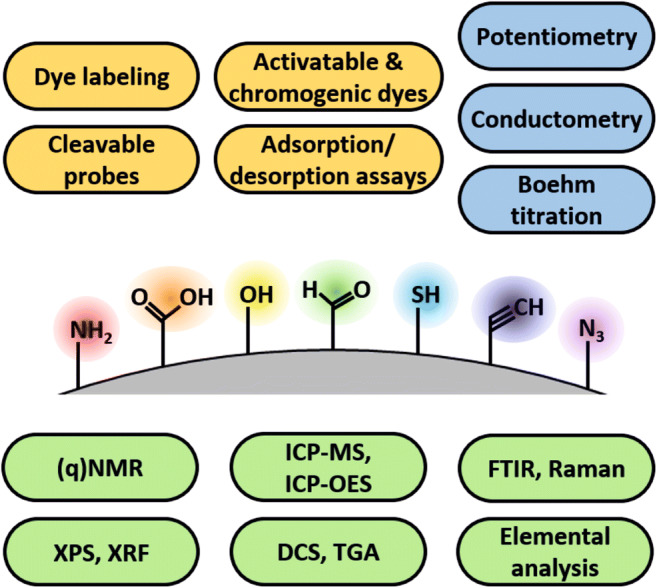

## Introduction

### Need for and importance of functional group quantification

Functionalized nanomaterials (NM) are of increasing industrial and economic importance in the life sciences and the health sector as well as for applications in nano(bio)technology, optical and sensor technologies, solid state lighting and photovoltaics, as well as opto-electronic and electronic devices and security applications. Nowadays, NM are used as catalysts, hydrogen storage and energy conversion materials, contrast agents and drug carriers for imaging and therapy in medicine, signal-generating reporters in bioanalysis, molecular diagnostics and sensing, as additives for food and cosmetics, in textile industry, and as phosphors for lighting and display technologies [1–13]. This comprises all types of core and core/shell NM such as organic polymer and inorganic silica or silica-coated nanoparticles (NP) with covalently bound surface groups as well as other inorganic NP like metal and metal oxide NP, semiconductor quantum dots (QD), and lanthanide-based NP with coordinatively or electrostatically bound ligands [14–16]. It also includes different types of encapsulated nanostructures like inorganic NP with hydrophobic surface ligands wrapped with amphiphilic (co)polymers or lipid coatings that can also be crosslinked, yielding micellar-type systems, or coated with alternating layers of differently charged polyelectrolytes by the so-called layer-by-layer (LbL) approach [17–19].

Decisive for most applications of NM are their specific surface properties, which are largely controlled by the chemical nature and number of ligands and functional group (FG) on the NM surface. The surface chemistry and surface FG determine the charge, dispersibility, and colloidal stability of NM, as well as their hydrophilicity/hydrophobicity, processability, and interaction with their environment [15, 18, 20–23]. In addition, FG enable the controlled modification and functionalization of NM by covalent binding of functional molecules such as hydrophilic ligands, anti-fouling agents, sensor dyes, and biomolecules like proteins, peptides, or oligonucleotides, e.g., for the preparation of nanosensors and targeted nanoprobes [5, 10, 24–26]. Control of the surface chemistry is also relevant for the minimization of unspecific adsorption, increase of colloidal and/or dissolution stability, and the design of drug carriers and triggered release systems [15, 22, 27–30]. For example, the reactivity and stability of NM can be altered intentionally and rationally by surface passivation strategies utilizing special coatings such as silica or polymeric shells, or via tailored modifications of the surface charge via the density of FG and ligands. This underlines the crucial importance of surface chemistry and surface functionalities for many NM applications in the life and material sciences and nano(bio)technology and their relevance for the rational design and tuning of the properties of functional NM. Knowledge of NM surface chemistry presents not only a key issue to understand the nano-bio interface largely controlling NM functionality and performance in (bio)applications, but is also relevant to assess the fate, exposure, dissolution, transformation, and accumulation of NM, and thus, NM toxicity and potential risks for human health and the environment [31–36]. This also includes the evaluation of risks associated with the application of engineered NM in consumer, food, and biomedical products [37–41]. Here, also unintentional changes and modifications in NM surface by time- and environment-dependent aging effects and transformations during the material’s life-cycle must be considered, that can affect NM safety aspects [42]. This is addressed by the increasingly pursued safe-by-design (SbD) concept of NM, which integrates considerations of material safety and performance as early as possible into the innovation process [43–45], thereby balancing safety, functionality, and costs for the development of better nanotechnology-enabled products throughout their life-cycle [46].

The increasing importance of NM in fundamental research and technological applications makes the sustainable development of functional and safe(r) NM as well as a comprehensive understanding of the structure-function and structure-safety relationships mandatory [43, 47]. Reliable, robust, and simple methods for the adequate characterization of such materials are key requirements to overcome challenges associated with the rapidly diversifying development of NM and to address still existing uncertainties and knowledge gaps [48, 49]. This is also essential for quality assurance and production control of engineered NM in support of the SbD concept [9, 18, 23, 50, 51]. In this context, the development of harmonized and standardized characterization methods not only simplifies to rank, prioritize, and choose safer alternatives during the innovation process of engineering NM, but is similarly beneficial for regulatory frameworks and the confidence in NM [41, 46, 52, 53]. Nevertheless, there is still a lack of normative measurement and characterization regulations, validated and standardized measurement protocols, reference materials of known surface chemistry for FG quantification, and reference data on application-relevant NM. Together with the often contradictorily literature in this field, this presents growing technological and economic challenges for manufacturers and users of NM. This has been increasingly recognized not only by scientists from different disciplines all over the world, but also by European legislation as well as national and international standardization organizations like ISO, IEC, and OECD [50, 54]. This makes the assessment of analytical methods for FG analysis on NM, including the determination of method-inherent limitations and NM-specific requirements and limitations as well as achievable method uncertainties, an increasingly important topic for NM-based technologies and NM risk assessment and regulation.

### Nanomaterial surface functionalization and bioanalytically relevant FG

The reactive FG and surface ligands relevant for NM-based (bio)analytical applications typically correspond with the complementary FG on the functional molecules utilized in typical (bio)conjugation reactions, e.g., biomolecules used as target-specific recognition moieties [24, 26, 55]. For biomolecules like peptides and proteins including antibodies and enzymes, this includes amino groups (-NH_2_) at the N-terminus of the polyamide backbone and at the side chains of the amino acids arginine, histidine, lysine, and tryptophan, carboxy groups (-COOH) at the C-terminus of the polyamide backbone and at the side chains of aspartic acid and glutamic acid, the hydroxyl and phenol groups (-OH) of serine, threonine, and tyrosine, the thiol (-SH) and thioether groups of cysteine and methionine, as well as chemically introduced thiol groups, e.g., via reductive cleavage of disulfide bridges [56, 57]. For carbohydrates like mono- and disaccharides, oligo- and polysaccharides, and as part of glycolipids and glycoproteins, the most abundant native FG are hydroxyl groups. Some monosaccharide derivatives such as non-acetylated amino sugars provide additional reactive FG for bioconjugation. Also, vicinal diols can be oxidized to aldehyde groups (-CHO). Oligonucleotides like DNA and RNA consist of a sugar-phosphate polymer backbone with a reactive phosphate group at the 5′-terminus and a OH group in the case of DNA or a vicinal diol for RNA at the 3′-terminus, respectively. The native nucleic acids cannot be chemically modified as easily as the amino acids in proteins. However, synthetic oligonucleotides can be prepared via solid-phase synthesis with aminoalkyl- or thioalkyl-containing linkers attached to the nucleobases, the phosphate backbone, or the 3′- or 5′-terminus, which allow for further modifications and labeling [58]. Besides native FG, there are several FG which can also be chemically introduced into biomolecules that are suitable for chemoselective labeling and bio-orthogonal chemistry such as azide (-N_3_), alkyne (-C≡CH), or maleimide groups for cycloaddition reactions [26, 57–59]. Other interesting FG for (bio)analytical applications are silanol (≡Si-OH, =Si(OH)_2_) and siloxane (≡Si-O-Si≡) groups present on silica particles and silica-based surface modifications and coatings used for all types of NM to improve or tune their stability and dispersibility in aqueous media, for SbD concepts and the supply of surface FG for further functionalization reactions [60–62].

### Quantifying total vs. derivatizable FG on nanomaterials

For the quantification of FG on NM, it needs to be distinguished between methods that provide the total number of FG present on the NM surface, and methods that determine the number of derivatizable FG [63, 64]. The total FG number largely determines NM charge (zeta potential), and thus, colloidal stability, dispersibility, and hydrophobicity/ hydrophilicity, and is therefore an important and application-relevant parameter for all types of NM in addition to size, size distribution, and shape/morphology. The number of derivatizable FG, in turn, controls the number of groups available for covalent attachment of functional molecules such as hydrophilic ligands, anti-fouling agents, or biomolecules. Hence, the number of derivatizable FG is important for all (bio)labeling reactions as well as the functionality and performance of the resulting surface-modified NP and NP (bio)conjugates.

**Fig. 1:**
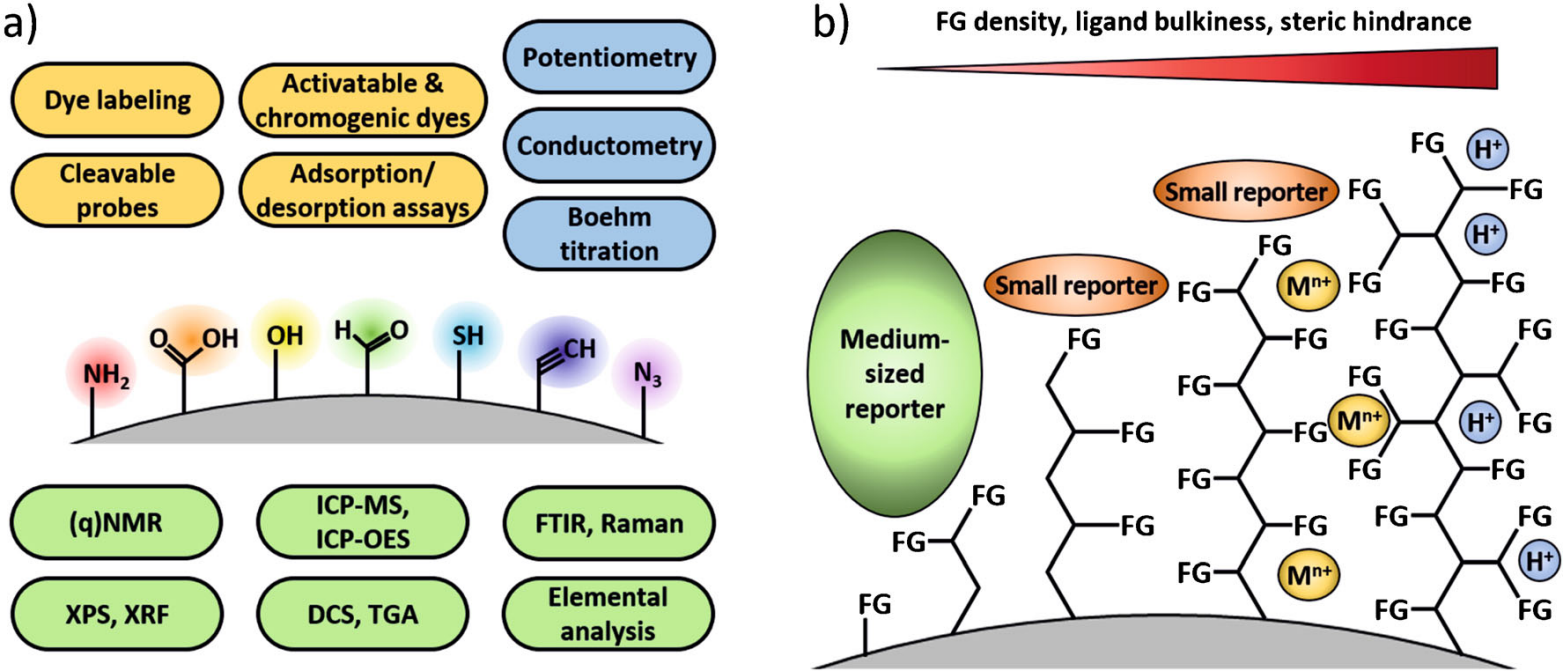
Brief overview of the bioanalytically relevant FG and the analytical methods covered by this review including typical reporters. **a** Method classification according to the principle of signal generation, i.e., electrochemical methods (blue), dye-based optical methods (yellow), and other instrumental analytical techniques (green). **b** Schematic presentation of the influence of the reporter size used to determine the number of FG depending on FG density or ligand bulkiness (steric hindrance) on the NP surface. The sizes of the labels can range from very small reporters like protons (H^+^) and metal (M^n+^) ions, to small and medium-sized reporters like organic dyes, that are still smaller than large biomolecules

Relevant for the selection of suitable analytical methods for FG and surface ligand quantification is the signal generation principle, i.e., whether these methods can quantify FG directly without a signal generating reporter (label-free methods), or whether they require a reporter for readout (label-based methods) that is covalently bound or interacts with the FG via electrostatic or adsorptive interactions [51]. In Fig. 1a, the analytical methods for FG quantification covered by this review are displayed and highlighted according to the principle of signal generation including electrochemical methods, dye-based optical methods, and other instrumental analytical techniques. For label-based methods, reporter properties such as molecule size, shape, and charge as well as the coupling efficiency and yield of the chemical reaction used for reporter conjugation can influence the analytical result. Especially the size and shape of the reporter utilized to determine the number of derivatizable FG can play an important role, as the obtained labeling density, and hence, number of derivatizable FG can be affected by the bulkiness of the label and steric effects [63], as shown in Fig. 1b. This, however, is also the case for any other covalently bound (bio)molecule of interest, so that the results can be correlated if a suitable reporter is applied, or can at least be estimated from the size and shape of the reporter and the (bio)molecule of interest. Moreover, it must be distinguished between absolute, i.e., calibration-free analytical methods and methods that require a calibration for analyte quantification. The latter is by far more common but can introduce additional challenges and uncertainties due to the need of a suitable reference material or standards, particularly for optical methods like absorption and fluorescence that yield signals which are affected by reporter environment. Depending on the method used, this can also determine whether the whole nanoobject can be analyzed as prepared, or whether the NM has to be dissolved prior to FG quantification.

Also, the type of bond between the NM and the FG-bearing ligand must be considered. For FG tightly bound to NP via covalently attached ligands, only steric effects affect the accessibility of the FG. For silica NP, it must be kept in mind that not all hydroxy groups are quantitatively involved in the grafting procedure and free ethoxy or methoxy groups remain on the silica NM. For electrostatically or coordinatively bound ligands, in turn, excess ligands must be removed and potential influences of NP concentration and dilution steps that can shift the ligand adsorption/desorption equilibrium must be excluded or properly considered for FG analysis [65]. For NP encapsulated in micellar structures, as often utilized for inorganic NP like iron oxide, semiconductor QD, and lanthanide-based upconversion NP [17, 18, 66, 67], even the orientation of the FG can play a role for FG analysis, depending on the chemical nature of the respective organic coating. Such systems can consist of buried FG pointing inwards that interact with the surface atoms of the encapsulated NP, and FG pointing outwards to the NP microenvironment. Only the latter ones are relevant for the covalent attachment of functional molecules. For such nanostructures, the information provided by the applied analytical method must be carefully evaluated regarding its information content to decide whether the whole nanoobject should be analyzed as prepared or whether the encapsulated NM should be dissolved prior to FG quantification.

Besides FG/ligand concentration, also the NM number concentration and surface area of the NM are of importance, as they determine the number of FG or ligands per particle as well as the FG or ligand density on the NM. Typically, the measured number of FG/ligands is divided by the number of particles or their total surface area. The NP number concentration can be determined via counting methods such as resistive pulse sensing (Coulter counter), nanoparticle tracking analysis (NTA), flow cytometry (FCM), as well as by absorption spectroscopy (if the molar absorption coefficient or the molar absorption cross section is known). The mean particle concentration can also be calculated indirectly from the NM mass (dry weight) or the concentration of certain NM-specific elements that can be quantified, e.g., with inductively coupled plasma mass spectrometry (ICP-MS) or optical emission spectroscopy (ICP-OES). The surface area, in turn, can be directly measured using gas sorption methods, or can be calculated from the NP concentration and the particle dimensions, obtained by sizing techniques such as transmission or scanning electron microscopy (TEM/SEM), small-angle X-ray scattering (SAXS), NTA, or dynamic light scattering (DLS). All these NM characterization techniques regarding NP size and concentration are well-known and have been reviewed in the literature [68–72], and we will focus here solely on methods for FG and ligand quantification.

For the determination of the total and derivatizable number of FG or ligands per particle, always another parameter needs to be considered, namely the distribution of NM size (and shape) and the corresponding variations of the surface-to-volume ratios within one particle batch, which directly influence the total surface area of the NM, and thus, the determined FG/ligand density (number of FG/ligands per particle). A perfect FG quantification method should be able to count the groups/ligands of interest per particle for a large number of individual particles to yield a histogram of FG or ligands per NM independent of particle size/size distribution and shape. A few sophisticated analytical techniques such as single-particle ICP-MS (sp-ICP-MS) or flow methods like FCM are in principle capable of measuring NM properties like elemental composition (for suited elements) and scattering and fluorescence (intensity) features on a particle-by-particle basis. These methods, however, still face limitations in NM surface characterization, e.g., related to the lack of sensitivity or influences of labeling chemistries (vide infra). Even if FG/ligand counting on single particles was possible, different morphological features (i.e., exposed crystal facets, local curvature radii of the surface) that can be present even at various surface areas of a single particle will still impact the ligand density on the NM surface. Thus, most analytical techniques applied for FG/ligand quantification and described here are ensemble techniques that yield only a mean value of FG/ligands for a given (mean) particle size and shape. As smaller particles have a smaller surface area, but a larger surface-to-volume ratio compared with larger particles, the distribution of FG/ligands per (individual) particles can strongly differ for particles with the same (mean) size but different size distributions. However, as the particle size has to be determined to calculate the surface area (vide supra), the obtained number-based size distribution can also be used to calculate the distribution of FG/ligands (assuming a similar surface morphology) and even to consider the size- and shape-dependent curvature of the NM surface.

In this review, typical methods for FG quantification on NM are presented (cf. Fig. 1a), and their working principles, advantages, and limitations are described, focusing on publications from the last 5–10 years and selected, representative examples to underline the versatility of the respective methods. Analytical techniques covered include electrochemical titration methods, optical assays, nuclear magnetic resonance (NMR), ICP-MS and ICP-OES, infrared (IR) and Raman spectroscopy, X-ray photoelectron spectroscopy (XPS) and X-ray fluorescence spectroscopy (XRF), as well as thermal analysis methods and elemental analysis. Other mass spectrometry techniques like laser ablation ICP-MS (LA-ICP-MS) or time-of-flight secondary ion mass spectrometry (ToF-SIMS) are not considered here, as these techniques are commonly used for the surface analysis of 2D-supports. Parameters addressed and used for method classification and evaluation include whether the respective analytical method (i) provides the total or derivatizable number of FG and (ii) is label-free or requires a signal-generating reporter; (iii) whether the reporter is covalently bound to the FG, giving rise to a possible influence of the efficiency of the conjugation reaction; (iv) the influence of reporter size; and (v) the need for method calibration. Special emphasis is dedicated to simple FG quantification methods with inexpensive instrumentation that are broadly accessible and can be used for routine analysis and process control during NM production and surface functionalization, like electrochemical and optical methods. We do not intend to cover NM bioconjugation strategies utilized for preparing nano-bioconjugates nor methods to quantify NM-bound biomolecules or to assess biomolecule functionality, which have already been excellently described in other review articles [56, 57].

## Electrochemical titrations for the quantification of (de)protonable FG on dispersed nanomaterials

Electrochemical titrations methods like potentiometric titrations (measurement of the electrochemical potential(s) of the sample), conductometric titrations (measurement of the sample conductivity), and the so-called Boehm titration that is specifically employed for carbon materials are commonly used as inexpensive and precise methods for the quantification of (de)protonable surface FG such as carboxylic acids, amines, or thiols. Closely related Zeta potential measurements that provide NM surface charge, which presents a measure for colloidal stability and can be used for the monitoring of reactions on nano- and microparticles [73], are not further detailed here.

During the course of an electrochemical titration, defined amounts of a titrant (typically acids or bases) are added to the sample, and the resulting changes in the electrochemical properties of the sample are monitored. As electrochemical acid-base titrations are typically carried out over a broad pH range, often including the isoelectric point of the sample (i.e., the pH value at which the net surface charge is zero), they can only be applied for NM that are stable under the given pH conditions. For example, the determination of amino groups (p*K*_a_ about 9–11) on silica-based NM can be challenging as silica dissolves at basic pH values, and some metal oxide and other chalcogenide-based NM can dissolve at acidic pH values where carboxylic groups (p*K*_a_ about 5) are typically detected. Nevertheless, for many other NM like carbon-based and polymeric particles, electrochemical titrations are well suited for FG and ligand quantification. Another limitation of electrochemical methods is their lack of specificity and selectivity, as all (de)protonable species with comparable p*K*_a_ values are detected which can distort the obtained results. This can include surfactants, initiators, or stabilizers from the NM synthesis, excess ligands with the (de)protonable group of interest, or the presence of other (de)protonable FG having a similar p*K*_a_ value. In addition, electrochemical titration methods require a relatively large amount of sample (typically about 10–20 mg/mL of NM sample).

### Potentiometric titration

**Fig. 2:**
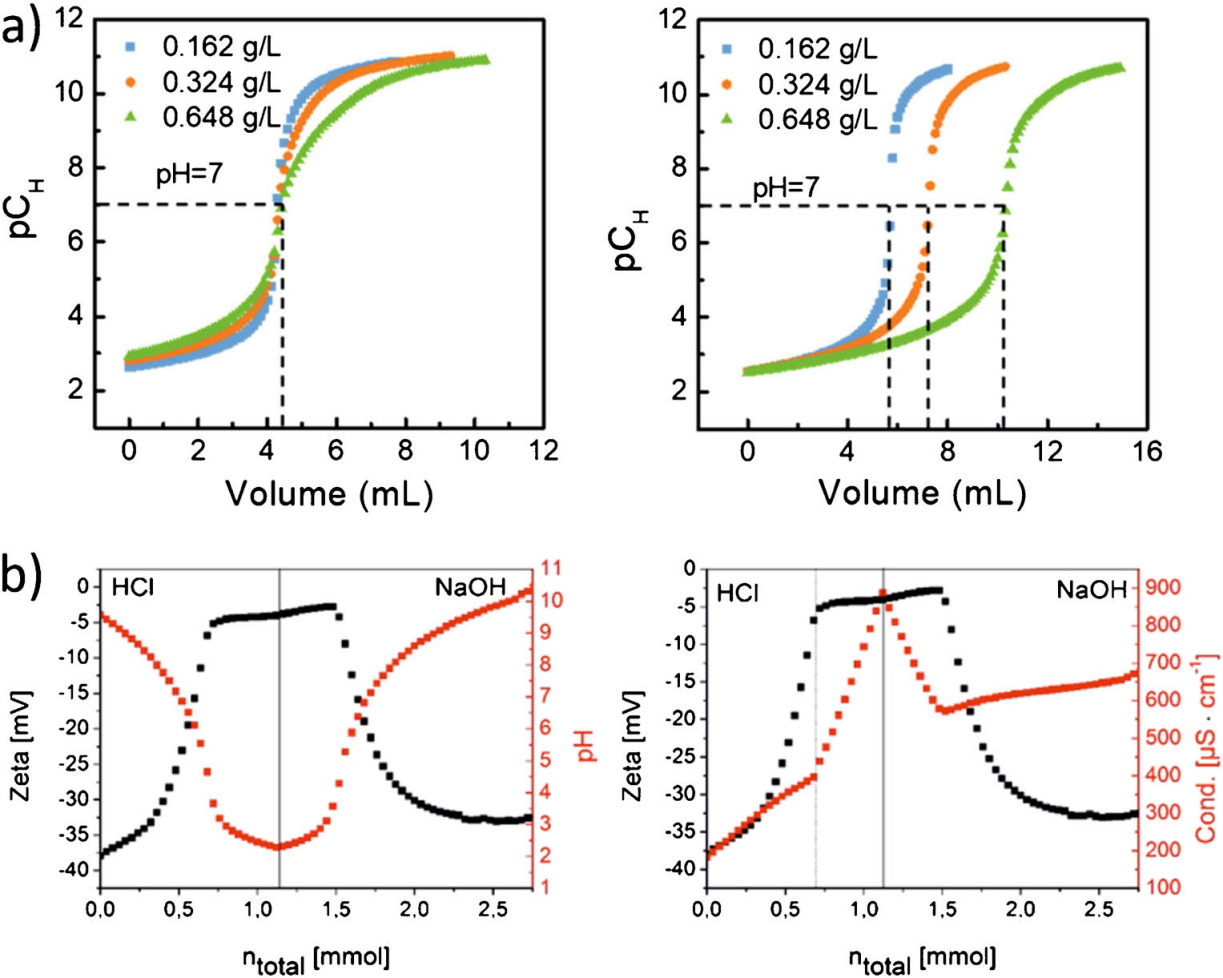
Representative examples for the FG quantification on NM using potentiometric titrations. **a** Results for the potentiometric FG quantification for carbon dots functionalized with different concentrations of either l-arginine (left) or l-glycine (right) using NaOH as titrant. Adapted with permission from ref. [87]. Copyright 2019, American Chemical Society. **b** Reversible deprotonation of a colloidal silica dispersion using HCl/NaOH titrants as detected by zeta potential (black) and pH (red, left) or conductivity (red, right) measurements. Adapted with author permission from ref. [88] (CC BY-NC 4.0)

In a potentiometric titration, the electrochemical potential(s) of the analyte solution is measured with two electrodes, normally in the form of pH measurements, upon addition of defined amounts of acid or base as titrant which yields a pH titration curve. The equivalence points of the titration curves provide the amount and the p*K*_a_ values of the (de)protonable FG of the analyzed sample. Also, the use of other ion-selective electrodes is possible [74, 75]. Potentiometric titrations have been used for characterizing the surface chemistry of different types of organic and inorganic NM with various FG, and have been applied to determine the number and nature of acidic sites (carboxy, lactone, phenol, and ester groups) on carbon-based materials like carbon dots (CD), nanocellulose/nanobentonite composites, biochar particles, multi-walled carbon nanotubes, or cellulose nanocrystals [76–80], or to quantify hydroxy (silanol) and thiol groups on hybrid silica particles [81]. Potentiometric titrations have also been used to determine the total number of acidic sites on different catalyst materials like phosphotungstic acid-functionalized Sn-TiO_2_ and organic-inorganic polyoxometalate NP, SrTiO_3_ particles used to catalyze condensation, hydrogenation, and amination reactions, functionalized silica particles employed as catalysts for the esterification of linoleic acid, and photocatalytic TiO_2_/S-doped carbon hybrids [82–86]. For example, Wang et al. potentiometrically quantified carboxy and amino groups on fluorescent CD prepared with different amounts of l-arginine or l-glycine (see Fig. 2a) [87]. By addition of Fe(III) ions before the titration and comparison of the results with measurements done without metal ions, the authors could also derive information on metal ion-CD interactions. Renner et al. compared potentiometric pH titrations with Zeta potential and conductivity measurements to quantify the number of hydroxy groups on silica and iron oxide NP [88]. The results obtained for silica particles, shown in Fig. 2b, demonstrate that Zeta potential values are closely linked to pH and conductivity of a sample, which is reflected by the respective curves changing at the same titrant volumes added. The titration with HCl used to protonate surface hydroxy groups was reversed by back titration with NaOH to ensure the reversibility of the process. The slightly negative zeta potential at full protonation was attributed to non-accessible hydroxy groups.

### Conductometric titration

In a conductometric titration, the conductivity of a sample is measured as a function of the added amount of acid or base. Typical examples present the quantification of the total amount of amino and carboxy groups on polystyrene (PS) particles [64, 89, 90]. The suitability of conductometry for carboxy group quantification on polymeric particles has been validated for polymethylmethacrylate (PMMA) particles grafted with polyacrylic acid (PAA) by comparison with quantitative NMR spectroscopy (qNMR) [63] and for PS particles by comparison with Zeta potential measurements [90]. Conductometric titrations are also commonly used for quantifying sulfate half-esters as well as carboxy and amino groups on cellulose nanocrystals (CNC), e.g., to achieve a tailored surface charge and to control CNC surface modification, or to study the effect of surface treatment on the dispersion rheology of CNC [91–95]. Other groups applied conductometric titrations to characterize CNC regarding their applicability for acid-base organo-catalysis [96, 97], or to determine the hydroxy group content on the surface of hydrogels consisting of modified cellulose nanofibrils suitable for controlled and pH-responsive release of a chemotherapeutic agent [98]. A general procedure for the determination of the sulfate half-ester content on CNC via conductometric titration, consisting of dialysis followed by treatment with a strong acid to ensure full protonation, was developed by Beck et al. [99], and a protocol to for the conductometric quantification of the sulfur and sulfate half-ester content on CNC was validated in an interlaboratory comparison [100]. The difference in the sulfur content determined by conductometry and by ICP-OES was attributed to sulfur in the CNC interior that is not conductometrically accessible.

### Boehm titration

**Fig. 3:**
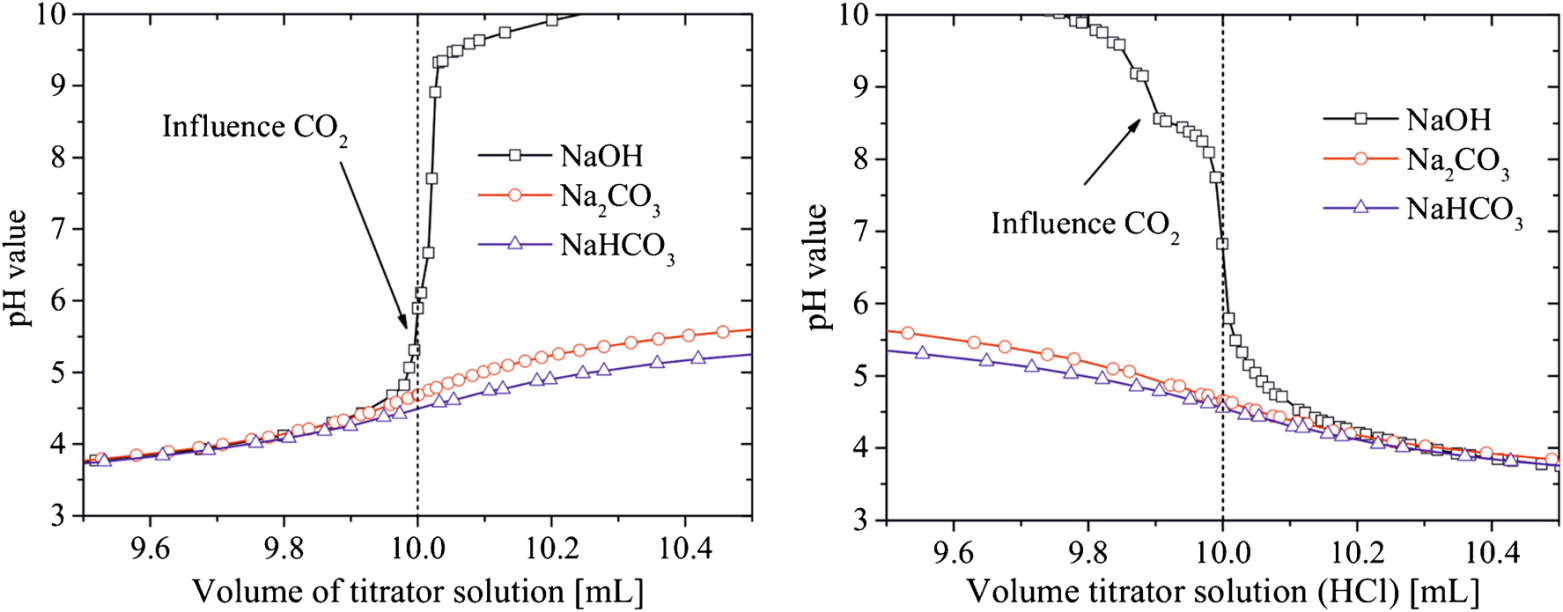
Boehm titration curves (potentiometric detection) obtained for the direct titration (**left**) and indirect titration (**right**) with HCl as analyte solution and NaHCO_3_, Na_2_CO_3_, or NaOH as titrant solutions, underlining the strong impact of CO_2_ from air on the results of the direct approach. Adapted from ref. [105] (CC BY 4.0)

Boehm titration is a method developed by Boehm et al. in 1964 [101] suitable for the quantification of acidic, oxygen-containing surface groups on various carbon-based materials such as graphene, carbon nanotubes (CNT), CD, and carbon-coated particles. This method allows not only to quantify common FG relevant for biolabeling and bioanalytical applications, but also other FG such as lactone or phenol groups [101–104]. The Boehm method is based on the treatment of a dispersed carbon sample with titration bases of different p*K*_a_ values like NaOH, Na_2_CO_3_, and NaHCO_3_ [101, 105], followed by the back titration of the unconsumed amount of the titrant. It is assumed that each base only neutralizes FG that are more acidic than the respective base, and the ratios of the amounts of the different oxygen-containing FG can then be calculated directly from the base consumption. Boehm titration has been utilized to characterize the FG on different kinds of CNT [106–108] as well as on other carbon-based materials like ozone-treated nanodiamonds, carbon NP derived from organic resin, graphite-decorated MnFe_2_O_4_ nanocomposites, and natural char nano- and microparticles [109–112]. To evaluate and standardize Boehm titration regarding accuracy, robustness, repeatability, and precision, Schönherr et al. investigated the FG on oxidized multi-walled CNT using different reaction bases, treatment times, and amounts of carbon material [105, 113]. A major concern of these studies was the dissolution of CO_2_ from air which leads to the formation of HCO_3_^−^ and CO_3_^2−^, that can considerably influence the titration results. To quantify this effect, a direct and an indirect approach to the Boehm titration procedure were compared. The results, shown in Fig. 3, underline the influence of CO_2_ particularly for the direct titration curve with NaOH. To circumvent such distortions, a medium-strong base like Na_2_CO_3_ was proposed and a protocol for an indirect titration approach with this base using an autotitrator was developed. Schönherr et al. also compared Boehm titration to other analytical techniques suitable for the quantification of oxygen-containing FG like XPS or temperature-programmed desorption mass spectrometry (TPD-MS), underlining its superior precision [105, 113].

Boehm titration is presently the only electrochemical titration method considered by international standardization organizations like IEC TC 113: *Nanotechnology for Electrotechnical Products and Systems* for surface FG analysis and quantification in the currently evaluated standardization document 62607-6-13: *Nanomanufacturing – Key control characteristics – Part 6*-*13: Determination of Oxygen Functional Groups Content of Graphene Materials with Boehm titration method*. The main purpose of this document is to provide a standardized method for the determination of surface oxygen FG on graphene materials prepared by, e.g., oxidation-reduction method, solution-phase exfoliation, micro mechanical exfoliation, and organic synthesis using the Boehm titration method and to obtain quantitative information about the acidic oxides at the surface of graphene materials, including carboxy groups (also in the form of their cyclic anhydrides), lactone groups, hydroxyl groups and reactive carbonyl groups.

## FG quantification with photometric and fluorometric assays and different optical reporters

FG quantification with optical spectroscopy relies on the measurement of the absorption (spectrophotometry; photometric or colorimetric assay) or emission (fluorometry; fluorometric assay) of a dye label (also called reporter or probe). Typically, fluorometric measurements are considered more sensitive than photometric measurements, as emission can in principle be detected down to the single molecule level, while absorption measurements utilizing the Beer-Lambert law for quantification commonly require a higher reporter concentration, depending on the reporter’s molar absorption coefficient. The dye label is either covalently bound to the FG on the NM surface requiring a reporter with a complementary reactive group, or interacts with the FG electrostatically in the case of adsorption/desorption assays [63]. In all cases, only the number of derivatizable FG is obtained (see also Fig. 1), which can considerably differ from the total amount of FG particularly for higher FG densities or concentrations, as most dye labels are much larger than the FG to be quantified. To correlate the measured optical properties with label concentration, optical quantification always requires a calibration with a dye closely matching the optical reporter used for FG quantification since the signal relevant optical properties of most reporters are influenced by reporter environment. Calibration can be carried out either with the free (unbound) dye itself, the reacted (bound) dye, or with a model system consisting of the optical label bound to a molecule mimicking the NM surface chemistry, given that the absorption and emission features are closely matching those of the sample.

Optical assays for FG quantification on NM can be distorted by interferences originating from light scattering by the NM, which in turn depends on NM size, excitation wavelength, and the difference in refractive index between the NM and its environment, as well as from NM absorption and/or emission. Only for very small NM (< 25 nm), light scattering is negligible and optical reporters bound to the NM surface can be quantified directly, if the NM does not absorb/emit at the same wavelengths as the dye reporter (spectral discrimination) and if dye-dye interactions at the NM surface can be excluded. For larger particles, light scattering can hamper a reliable and accurate quantification in the presence of the NM. In these cases, the NM has to be removed prior to optical dye quantification by either filtration or centrifugation. Alternatively, the particles must be dissolved, so that only the reporter dyes present in the transparent solution are detected. Also, FG determination via optical reporters can be done by an indirect quantification of unbound labels, or with the aid of cleavable probes and catch-and-release assays where the optical reporter is readout in a transparent solution as detailed in the following sections.

**Fig. 4:**
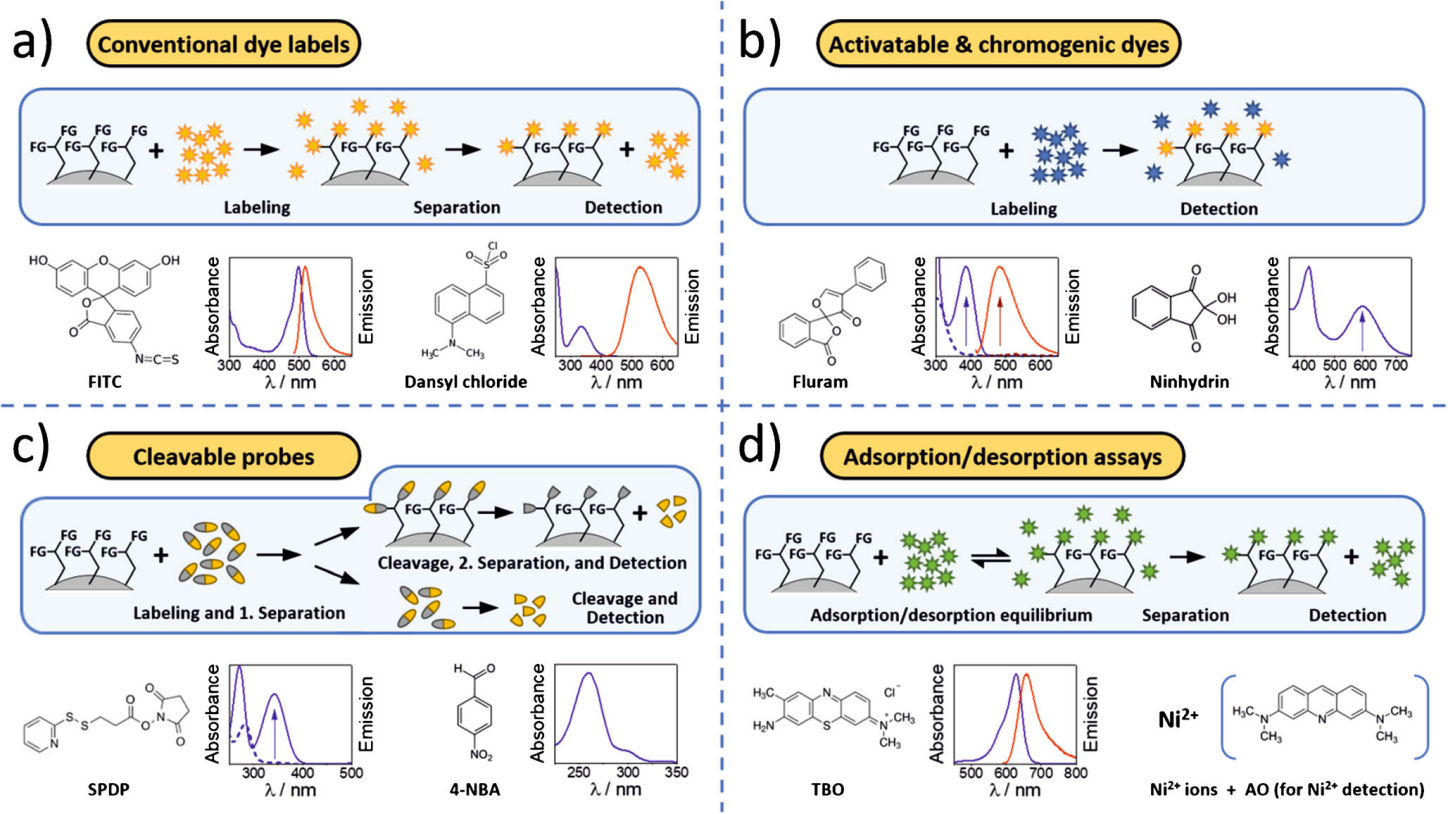
Schematic presentation of the working principles of different photometric and/or fluorometric assays for FG quantification on NM using different optical reporters including typical examples for respective dye-based reporters and their absorption and/or emission spectra

A broad variety of optical assays for different FG on NM and 2D-supports has been developed which utilize different types of absorbing or fluorescent labels. As summarized in Fig. 4, this includes (i) conventional (“always ON”) dyes, (ii) chromogenic/fluorogenic (“chameleon”-type) dyes and activatable (“turn-ON”) dye reporters that change either the spectral position of their absorption and/or emission bands upon reaction with the respective FG or become absorptive (colored) or emissive upon the binding event [64, 114, 115], and (iii) cleavable probes that can be quantitatively cleaved off from the NM surface and subsequently quantified in solution [64, 89]. In addition, (iv) adsorption/desorption assays relying on negatively or positively charged reporters and electrostatic interactions with oppositely charged FG are utilized [116]. These different types of optical assays are subsequently described and compared including representative examples.

### FG quantification with conventional “always ON” dyes

Conventional dyes used for FG quantification on NM are fluorophores with a reactive group that allow for the covalent coupling of the label to the FG on the NM surface. Due to the large toolbox of commercial dyes available from different fluorophore classes bearing different reactive groups, that were developed for bioconjugation reactions ranging from simple NHS chemistry to biorthogonal reactions and Click chemistry, this approach can be utilized for all types of bioanalytically relevant FG. The optical properties of conventional dye labels such as the spectral position of their absorption and emission bands as well as their absorption and emission intensities, determined by their molar absorption coefficients and photoluminescence quantum yields (QY_PL_) commonly change only slightly upon NM conjugation. The size of such changes, particularly in QY_PL_, depend on dye class, the optical transitions involved, and on the length of the linker between the reactive group of the reporter binding to the NM surface and the dye’s chromophore system. This is advantageous and disadvantageous at the same time. As NM-bound dyes and unbound (free) dyes cannot be spectroscopically distinguished, a separation of bound and unbound dye molecules is necessary prior to optical quantification [64].

**Fig. 5:**
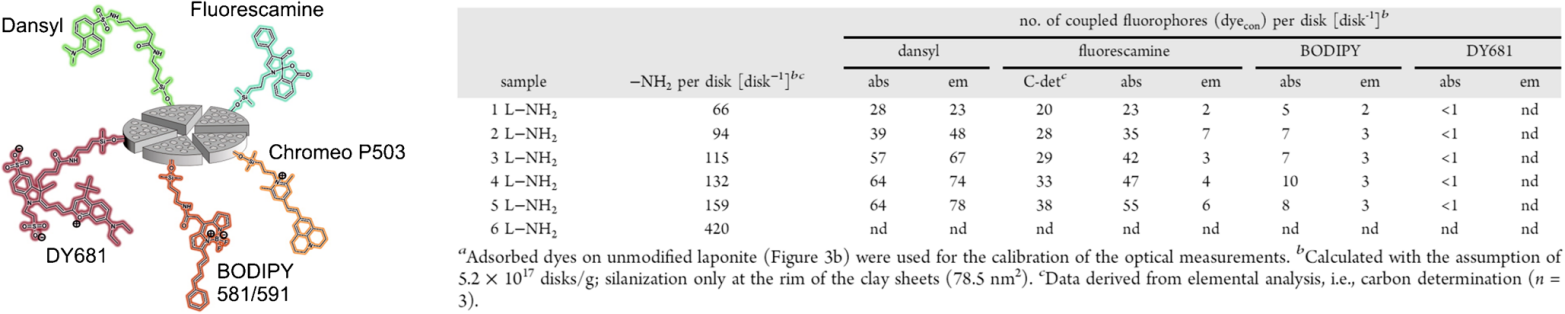
Quantification of the derivatizable amino groups on APTES-modified laponite disks using differently charged NHS-activated conventional dyes. Reprinted with permission from ref. [114]. Copyright 2015, American Chemical Society

Conventional dyes have been applied for the quantification of derivatizable FG on various kinds of inorganic, organic, and hybrid NM, e.g., amino groups on a silane surface using a self-made BODIPY dye and a commercial Rhodamine B dye [117], or aldehyde and azide-containing ligands on the surface of CdSe-ZnS QD using 2-hydrozinopyridine (forming a stable hydrazone chromophore with aldehydes) or an NHS-activated Cy3 dye in conjunction with amino-dibenzocyclooctyne crosslinkers, respectively [118]. Felbeck et al. utilized various NHS-activated conventional dyes to quantify amino groups on the surface of laponite nanoclays modified with 3-(aminopropyl)triethoxysilane (APTES), as shown in Fig. 5 [114]. The authors compared dyes of different charge like the negatively charged hemi-cyanine DY681, the zwitterionic BODIPY 581/591, a neutral dansyl derivative, and the positively charged pyrylium dye Chromeo P503. While charged dyes were prone to aggregation or did not react with the FG on the laponite surface due to electrostatic repulsion, the neutral dansyl dye enabled efficient labeling of the amino groups of APTES.

For conventional dye labels, an indirect quantification, i.e., the quantification of the amount of unbound dye molecules, is recommended as the most effective and reliable way to determine the number of accessible FG [64]. Depending on the NM, alternatively, the dye-functionalized sample can be dissolved after removal of unbound label, followed by optical quantification of the residual reporter molecules. Only if light scattering is negligible (e.g., due to a small NP size) and dye-dye interactions can be excluded (e.g., due to a low FG density on the NM surface), a direct quantification of the particle-bound reporters leads to reliable and accurate results [119]. A strategy to circumvent dye-dye interactions for higher FG densities presents NM labeling with a mixture of dye molecules and non-functional molecules bearing the same reactive group, thereby diluting the dye reporters at the NM surface [63]. In this case, FG quantification relies on the assumption of identical coupling efficiencies of both reactants, which needs to be validated individually.

### Activatable (“turn-ON”) and chromogenic (“chameleon”) reporter dyes

Activatable reporters are dye precursors that become strongly absorbing (“colored”) or emissive (so-called turn-ON dyes) after covalent coupling to the respective FG on the NM surface, while chromogenic dyes display significant spectral shifts in their absorption and/or emission bands upon the covalent attachment to FG. The latter dyes are sometimes also referred to as “chameleon dyes.” Well-known examples for activatable dyes utilized as reporters in photometric and fluorometric assays are Ninhydrin (2,2-dihydroxyindane-1,3-dione) and Fluram (4′-phenylspiro[2-benzofuran-3,2′-furan]-1,3′-dione) that both form optically detectable products upon reaction with primary amino groups.

**Fig. 6:**
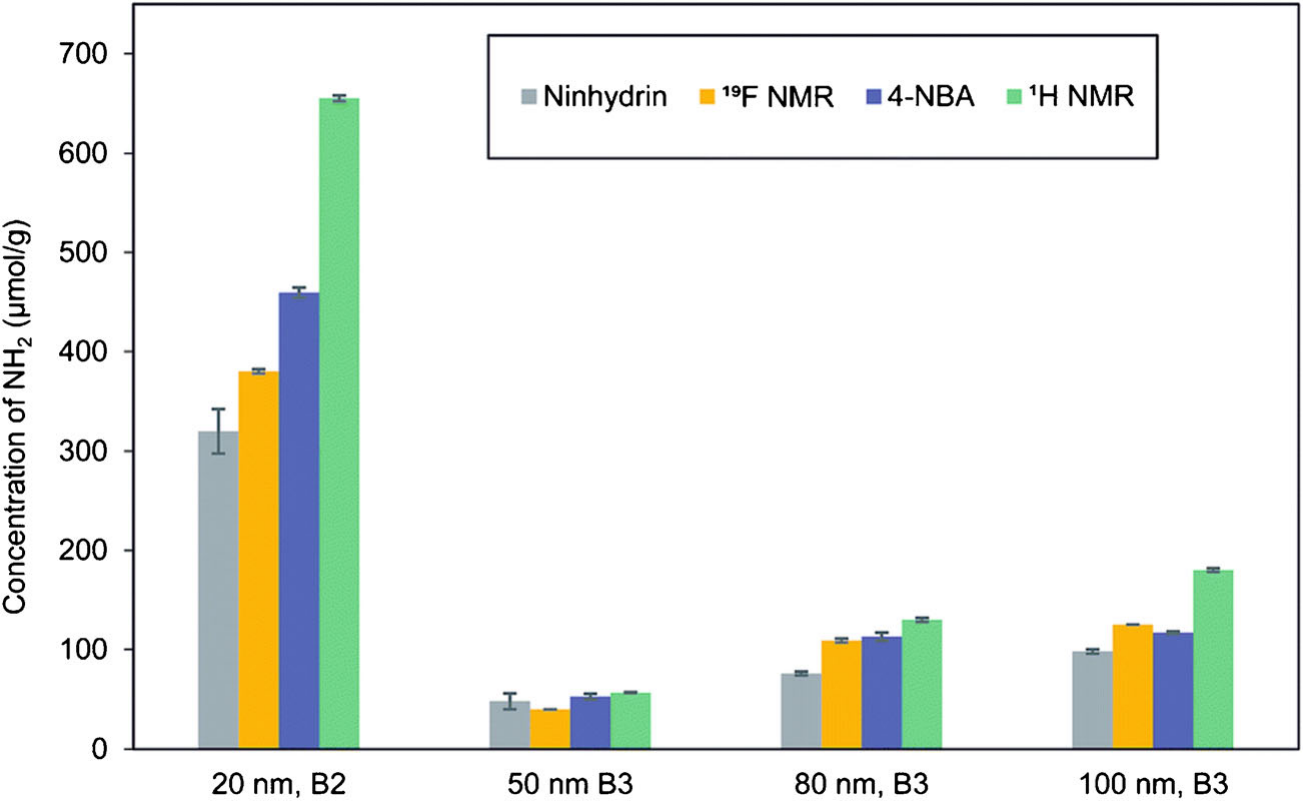
Comparison of two optical assays utilizing dye reporters (Ninhydrin, 4-nitrobenzaldehyde) and quantitative ^19^F NMR using the F-containing label trifluoromethyl benzaldehyde for FG quantification on amino-modified silica NP of different sizes. Reprinted from ref. [123] with permission from the authors (CC BY-NC 3.0)

Ninhydrin, that has been initially used in protein assays, forms the dye Ruhemann’s Purple with primary amino groups, absorbing at about 570 nm [120]. The photometrically detectable colored species is released and the absorption measurement is done in the supernatant, thereby circumventing interferences from possible scattering of the excitation light by the NM. As the Ninhydrin reaction is an equilibrium reaction, Ruhemann’s Purple is continuously generated in the presence of primary amino groups reacting to aldehyde functionalities, which reduces the impact of steric crowding of multiple Ninhydrin molecules occupying neighboring FG. Ninhydrin was, e.g., used to monitor the reproducibility of silica particle synthesis and their modification with APTES in comparison to NMR measurements [121], and to study the FG density and colloidal stability of surface functionalized silica NP over a period of time of 30 days [122]. Sun et al. compared the quantification potential of two optical assays (Ninhydrin and 4-nitrobenzaldehyde) with ^19^F solid state NMR measurements to quantify FG on amino-modified silica NP of different sizes (see Fig. 6) [123].

Fluram, that is also referred to as Fluorescamine, is a colorless dye precursor that forms a yellow product with primary amines with a strong emission between 400 and 600 nm. As the amino group is integrated into the fluorophore in a ring-formation mechanism, the emissive Fluram product needs to be measured directly bound to the NM surface. As unreacted Fluram itself is not emissive, no washing or purification steps are required to remove the unreacted precursor dye prior to assay readout. As the emissive dye product is of limited stability, the assay should be read out at a constant time point after the reaction with Fluram. Moreover, particle light scattering and dye-dye interactions can interfere with the Fluram assay, as has been demonstrated in comparison to the results obtained with the cleavable fluorenylmethyloxycarbonyl protecting group (Fmoc, vide infra) [124]. Fluram was applied to study the influence of the FG density on the biocompatibility of aminated silica NP [125], and to quantify primary amino groups on nanoclays (see Fig. 5) as well as on PS nano- and microparticles [64, 114]. In the latter case, a reliable and accurate FG quantification with the Fluram assay involved the dissolution of the polymer particles in an organic solvent and a correlation of the subsequently detected dye signal with a calibration curve obtained with a suitable model system consisting of the dye bound to a small molecule such as propylamine bearing a primary amino group.

A related method, here for the quantification of thiol groups, is the Ellman’s assay which exploits the reaction of 5,5′-dithiobis-2-nitrobenzoic acid (DTNB, also called Ellman’s reagent) with thiolate anions to a mixed disulfide and 2-nitro-5-thiobenzoic acid, which can be detected photometrically at about 410 nm. The Ellman’s assay, which has been initially developed for the quantification of thiol groups on proteins, has been used to quantify thiol groups (directly) or maleimide groups (indirectly after reaction with l-cysteine) on functionalized PS particles [126, 127], and to determine the number of thiol ligands like mercaptopropionic acid (MPA) or dithiol dihydrolipoic acid (DHLA) on semiconductor QD and noble metal particles [65, 127].

Chameleon dyes possess an electron-withdrawing group conjugated with the chromophore π-system (e.g., a halogen atom such as -Cl) that is transformed upon the reaction with a FG, e.g., a primary amino group, into an electron-donating group, resulting in strong blue shifts in absorption and emission. The spectral shifts as well as the changes in the molar absorption coefficients and QY_PL_ values are considerably influenced by the exact chemical structure of the analyte or FG (i.e., the electron donating amino group-containing ligand) substituting the electron-withdrawing group. Cyanine-based chameleon dyes have been utilized for the labeling and subsequent detection of biomolecules containing primary amino groups [128] and to confirm the amino modification of silica and PS NP both photometrically and fluorometrically [129]. The chameleon dye IR797 was used by us to quantify the amount of accessible amino groups on PS nano- and microbeads [64]. As for activatable reporters, a reliable and accurate FG quantification required the dissolution of the dye-bound particles and a thorough calibration with a suitable model system. Another class of chameleon dyes also suited for FG quantification are pyrylium reporters that react with primary amino groups to form pyridinium dyes with strongly blue shifted absorption and emission bands [115].

The advantage of activatable and chromogenic dye reporters compared to conventional dye labels are the different optical properties of the NM-bound and free dyes, allowing for a straightforward spectroscopic discrimination between these species. For activatable dyes, only a quantification of the NM-bound reporters is possible, which can be hampered by particle light scattering, while for chromogenic labels a spectroscopic quantification of both the NM-bound dyes and the unreacted free dyes is feasible. Moreover, some NM such as metal particles or semiconductor QD exhibit strong absorption and/or emission bands that can interfere with the dye spectra. As for conventional labels, FG quantification requires a calibration curve from a model system with absorption and/or emission properties that closely match those of the NM-bound activatable or chromogenic dyes to consider the environment dependence of the absorption and emission features of the reporter, particularly its QY_PL_.

### Labeling with cleavable probes, catch-and-release assays, and indicator displacement assays

Modularly built cleavable probes consist of a reactive group that can be coupled to the FG of interest, a cleavable linker that can be cleaved fast and quantitatively after the conjugation reaction, and a reporter unit subsequently released which can be quantified photometrically or fluorometrically. Suitable cleavable linkers can be taken from established drug release concepts like pH-cleavable hydrazone bonds or reductively cleavable disulfide bridges. Another type of cleavable probes are optically detectable protection groups like Fmoc, which is frequently used to determine resin substitution in solid-phase peptide synthesis. Fmoc can be used for the quantification of amino groups on NM by cleaving off the NM-bound Fmoc protecting groups with piperidine in DMF followed by photometric or fluorometric detection of the released dibenzofulvene-piperidine adduct [130–136]. Meanwhile, variations of the reaction solvent [137] and other suitable detection wavelengths [138] have been reported. To increase the sensitivity of the assays, that was initially read out photometrically, a Fmoc-Cl fluorescence assay was developed that can be performed in aqueous solution. This assay is approximately 50–200-fold more sensitive than the photometric method, but the separation of excess Fmoc-Cl and its strongly fluorescent reaction products is still challenging [124, 139].

**Fig. 7:**
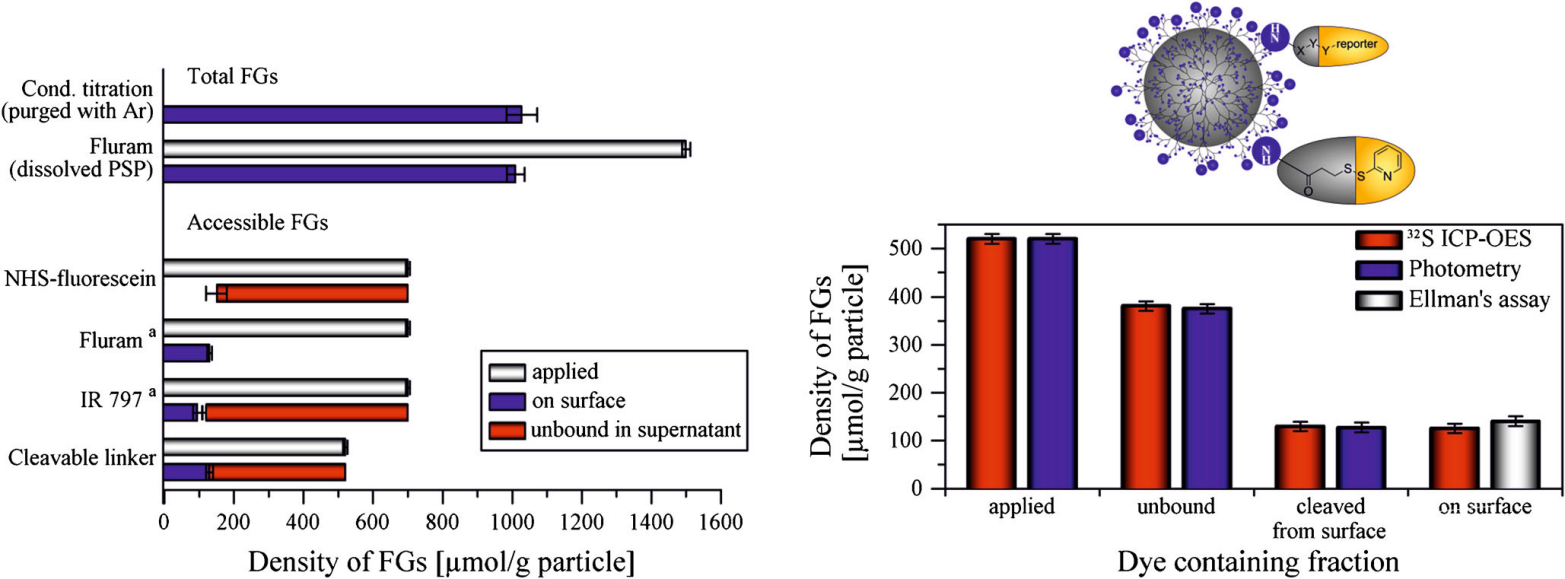
Comparison of optical FG quantification using cleavable probes, conventional dyes, and activatable/chromogenic reporters. Validation of the former approach was done with ICP-OES and the Ellman’s assay. Reprinted with permission from ref. [64]. Copyright 2018, American Chemical Society. Further permissions related to the material excerpted should be directed to the American Chemical Society

We rationally designed the cleavable probes *N*-succinimidyl-3-(2-pyridyldithio) propionate (SPDP) and *N*-(aminoethyl)-3-(pyridin-2-yldisulfanyl)-propane amide (*N*-APPA) by combining a reactive NHS- or amino group, a reductively cleavable disulfide linker, and a simple 2-thiopyridone reporter unit. [64] To demonstrate the advantages of these cleavable probes, we compared their performance with that of conventional dye labels and activatable/chromogenic reporter dyes (vide supra) for the quantification of amino and carboxy groups on PS nano- and microparticles, as shown in Fig. 7. This comparison confirmed the advantages of SPDP and *N*-APPA for FG quantification, i.e., the possibility for determining mass balances and a straightforward method validation with other analytical methods like ^32^S ICP-OES. These cleavable probes are also suited for quantifying amino and carboxy groups at various FG densities and even on absorbing and fluorescent NM like dye-stained fluorescent particles.[89]. Moreover, this design principle can be easily adapted to other FG. For example, we developed the SPDP derivative 3-(2-pyridyldithio)propionyl hydrazide (PDPH) bearing a reactive hydrazide group for the quantification of aldehyde groups on a set of PMMA microparticles [140]. Validation was done by comparison with another catch-and-release assay utilizing a hydrazide-functionalized fluorescent BODIPY dye (BDP-hzd) as reporter that proved to be even more sensitive due to the fluorometric readout.

Other examples for optical assays utilizing cleavable probes present the use of 7-methoxycoumarin-3-carboxylic acid conjugated to an alkyne group for the fluorometric quantification of azide groups on solid substrates, silica particles, and biomolecules after cleaving off the dye under basic conditions [141], and the use of 4-nitrobenzaldehyde (4-NBA) for the photometric quantification of amino groups on silica particles of different sizes (see Fig. 6) after dye hydrolysis [123]. A similar type of assay that also relies on reporter detection in solution after particle removal is the so-called indicator displacement assay that exploits supramolecular host-guest chemistry, e.g., the competitive binding of the reporter dye acridine orange (AO) and the high affinity guest aminomethyladamantane (AMADA) to the macrocycle host cucurbit[7]uril (CB7) for the optical quantification of azide groups on PMMA particles [142].

Cleavable probes and catch-and-release assays are ideally suited for FG quantification on NM, since quantification of both the unbound (unreacted) dye and the initially particle-bound reporter (after cleavage) can be performed in solution after removal of the NM. Hence, these methods allow for FG quantification without interferences from light scattering, absorbing and/or emitting NM, or dye-dye interactions of surface-bound reporters. The cleavable reporter approach enables to generate a mass balance from the known amount of applied label and the measured amount of unbound and bound reporters, which increases the accuracy and reliability of this FG quantification method and simplifies method validation. In addition, the modular design of the cleavable probes offers the opportunity to specifically choose the reactive group, the cleavable linker, and the reporter unit according to the desired application and sample-specific requirements (specific type of target FG, limitations due to particle material-related properties, etc.).

### Optical adsorption/desorption assays

Alternatives to optical assays involving covalent labeling are adsorption/desorption assays with photometric or fluorometric readout. In an adsorption/desorption assay, optically detectable reporter dyes or, less common, small metal ions with charges complementary to that of the FG of interest, are allowed to adsorb at the charged NM surface. The reporters must not bear a reactive group and should not penetrate the particle matrix. Subsequently, non-adsorbed probe molecules are removed by several washing steps, followed by quantitative desorption of the adsorbed reporter by addition of a surfactant. Then, the desorbed reporter is quantified photometrically or fluorometrically in the supernatant after removal of the NM by centrifugation or filtration. In the case of metal ions, with few exceptions [143], optical detection is achieved by addition of an indicator dye that forms a colored or fluorescent product of defined stoichiometry with the metal ion. Alternatively, the non-adsorbed amount of the reporter can be quantified after removal of the NM containing the fraction of the adsorbed reporter. In conjunction with a fluorescent dye and readout with fluorescence microscopy or flow cytometry, also the amount of particle-adsorbed fluorophore can be measured directly [144]. The use of metal ions as reporters in adsorption/desorption assays for charged FG like carboxy and amino groups exploits the much smaller size of metal ions compared to organic dyes which is expected to provide a reporter-to-FG stoichiometry close to 1, and thus, a number of (accessible) FG approaching the total number of FG.

A popular dye-based adsorption/desorption assay for the photometric quantification of carboxy groups is based on the cationic dye toluidine blue (TBO) that displays an intense blue color with an absorption maximum at around 630 nm [63]. An example for a dye-based adsorption/desorption assay with fluorometric detection utilizes the red emissive cyanine-type nucleic acid stain SYTO-62 [144]. Metal ion-based adsorption/desorption assays have been reported by several research groups, e.g., using Mg^2+^ ions to quantify tryptophan ligands on gold NP [143], or Ni^2+^ ions to quantify carboxy groups on polymeric microparticles [116, 145].

Advantages of adsorption/desorption assays are their simplicity, as they are in principle suitable for all types of NM bearing charged FG independent of their chemical composition, if dye penetration into the NM matrix can be excluded. Thus, such assays are not suited for porous materials like mesoporous silica NP. They are very versatile and require only one calibration for different NM samples. A drawback presents the time-consuming washing steps needed for quantitative dye desorption. Moreover, as typically more than one FG interacts with one reporter molecule, FG quantification requires the determination of a stoichiometry factor by comparison with the results obtained by another method yielding the total number of FG [63]. This stoichiometry factor is most likely NM-specific, which can affect the reliability of FG quantification with this type of assay without a thorough validation. Adsorption/desorption assays are well suited for quality assurance and process control (including control of product reproducibility) of NM bearing charged FG as well as the monitoring of aging effects affecting surface FG, as such conclusions can be drawn based upon relative comparisons.

## Other methods for FG quantification on nanomaterials

Other analytical techniques used for FG analysis and quantification on NM surfaces include NMR spectroscopy, ICP-MS and ICP-OES, IR and Raman spectroscopy, X-ray-based methods such as XPS and XRF, as well as thermal analysis methods and elemental analysis [18, 23, 51]. Depending on the chemical nature of the NM and the FG of interest, these analytical techniques can either utilize intrinsically present moieties (label-free methods) or specific reporters (label-based methods) for signal generation and quantification, and thereby, provide the total or derivatizable number of FG. These analytical methods are very valuable tools for FG quantification and for validation of the results obtained with simpler methods (method validation). A straightforward approach to simplify method comparisons for method validation and calibration is the utilization of multimodal labels and reporters that are designed for the readout by different analytical techniques relying on different signal generation principles. Multimodal reporters can be realized, e.g., by including heteroatoms like sulfur, nitrogen, fluorine, or certain metal ions into molecular labels like organic dyes used for chemical derivatization reactions or reporters utilized for the design of cleavable probes. In the following, also examples for this strategy including its use to provide a traceability chain of FG quantification to the SI unit mole are highlighted.

### Nuclear magnetic resonance spectroscopy

Nuclear magnetic resonance (NMR) spectroscopy measures the intrinsic magnetic moments of certain nuclei such as hydrogen (^1^H), carbon (^13^C), fluorine (^19^F), or phosphorus (^31^P) in the presence of a strong magnetic field. NMR spectroscopy can provide chemical, physical, and structural information about the NM, its organic ligand shell and surface FG, as well as information on dynamic interactions with the environment [146]. Moreover, it can distinguish between surface bound and free (excess) ligands which has been exploited to gain a deeper understanding of the NM-ligand interface including ligand binding sites and dynamics, particularly for semiconductor QD with their surface-dependent luminescence properties [29, 147–150].

The quantification of FG and ligands on NM surfaces can be carried out by solution phase NMR techniques and by solid-state NMR. It typically involves the addition of an internal standard of known concentration and known, high purity, that is chosen to reveal NMR signals (chemical shift) well separated from the NMR signals originating from the FG or ligands of interest and the matrix [51, 89]. Particularly for solution NMR, it must also be considered that the NMR signals of the FG or ligands bound to a NM surface can significantly change compared to the signals of the free molecule or FG in solution. The NMR signals obtained typically show differences in both chemical shift and linewidth due to homogeneous and/or inhomogeneous line broadening, which can hamper peak assignment and integration [151]. The size of such effects depends on NM size. Since the percent weight of the bound surface ligands decreases with increasing particle size, quantification of larger size NM can require a relatively large amount of sample compared to other analytical methods. To overcome these limitations, dissolution methods for FG and ligand quantification prior to NMR analysis have been developed [121, 152]. Particularly for organic polymer particles, the distinction of NMR signals originating from surface FG and the NM matrix can present a challenge, which can be met by using isotope-enriched reagents for surface functionalization [63], or by applying a multi-Lorentzian-splitting algorithm [153]. Also, combinations of multinuclear and multidimensional NMR techniques are a promising approach to identify and quantify FG/ligands, and to study their interaction with and binding to the NM surface [154–156].

Quantitative NMR (qNMR) is particularly attractive for FG quantification, due to its potential for an absolute quantification. Moreover, it can provide traceability to the SI unit mole if suitable calibration materials of very high and known purity are available [157, 158]. For instance, qNMR was used to study the FG or ligand density on gold NP [159, 160], semiconductor QDs [161], and silica NPs [123, 162]. However, qNMR requires special measurement conditions. Prior to collecting NMR spectra, the *T*_1_ relaxation times of the components must be determined with a series of inversion-recovery experiments. These *T*_1_ times then have to be considered for the recording of the NMR spectra used for signal quantification which commonly requires a relatively high number of scans, and thus, elongated measurement times. For data evaluation, the integral values of the evaluated signals must be baseline-corrected and the purity of the internal standard added in a precisely known amount must be determined, for example by comparing its NMR signals to that of a reference material of certified purity. Despite the need for expensive equipment operated by well-trained scientists, qNMR has become increasingly popular for the quantification of FG on NM due to its inherent chemical selectivity and the provision of the total number of FG without the need for a calibration curve. Also, this technique is increasingly used for the calibration and validation of other more simple analytical methods, particularly for NM where electrochemical titrations cannot be utilized due to interferences from the NM matrix, or for the quantification of FG that are electrochemically not accessible. For example, the quantification of carboxylate and amino functionalities on silica NM is not feasible by electrochemical titrations as the p*K*_a_ values of the inherently present silanol groups and carboxylate groups cannot be well separated and as silica NM dissolve at alkaline pH. Here, qNMR is the method of choice for the quantification of the total amount of carboxylate and amino groups [89, 123, 156, 163, 164].

**Fig. 8:**
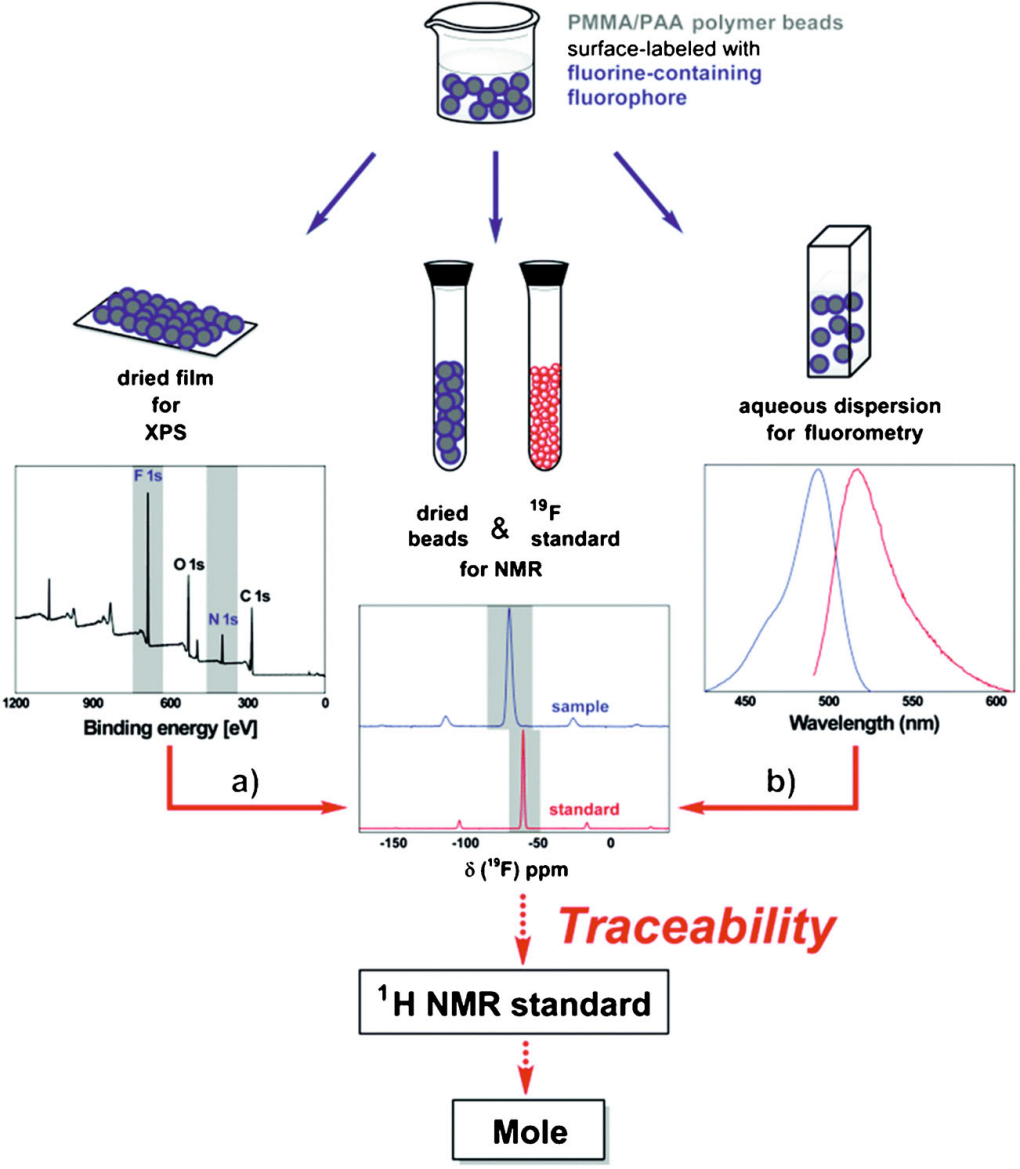
Example for a traceability chain for FG quantification, linking measurements (blue arrows) of XPS (**a**) and fluorometry (**b**) to quantitative solid-state ^19^F NMR (solid red arrows). The use of a certified NMR reference standard containing both ^19^F and ^1^H provides the link to the SI unit mole (dotted red arrows). Reprinted from Ref. [158] (CC BY 3.0 unported)

An example for a possible traceability chain for FG analysis with different analytical methods including NMR is shown in Fig. 8, using multimodal reporters that can be read out by different analytical techniques, here solid-state ^19^F NMR, emission spectroscopy, and XPS, and a certified NMR reference standard containing both ^19^F and ^1^H [157, 158].

### Mass spectrometry and atomic spectroscopy

Mass spectrometry (MS) and optical emission spectrometry (OES; also referred to as atomic emission spectroscopy, AES) can quantitatively measure the total number of atoms of certain elements within a sample. This can be utilized for the quantification of surface FG and ligands on NM as well as for the determination of NP concentration. Typically, both techniques use inductively coupled plasma (ICP) to produce excited atoms and ions, which are then identified based upon their characteristic mass-to-charge ratios (ICP-MS) or atomic spectral emission lines (ICP-OES). ICP-MS and ICP-OES directly measure the element concentration with unparalleled sensitivity over a wide linear dynamic range regardless of NM size or surface chemistry down to the parts-per-trillion level. However, the achievable detection limits (LOD) depend on the element(s) of interest, instrument, experimental conditions, and possible spectroscopic interferences between the analyte elements. For NM analysis, ICP-MS and ICP-OES are often used in connection with an upstream particle separation method like high performance liquid chromatography (HPLC) or asymmetric flow field-flow fractionation (AF4) [165–170].

ICP-MS and ICP-OES are often applied to determine the NM concentration from the measured element concentration of the sample in combination with the known NM dimensions, typically determined with sizing techniques such as TEM, SAXS, DLS, or NTA [166, 171, 172]. However, both methods can also be applied to detect elements that directly correspond to ligands and FG native on the NM surface, and thus, the total amount of FG, or to detect specific labels conjugated or associated to the FG, and hence, the number of derivatizable FG. Examples for the latter case present the quantification of elements such as sulfur present in certain dyes or cleavable probes [64, 89, 127], or the use of metal ion containing reporters [173]. ICP-OES has been used to quantify FG on various NM such as carbon-based NM, noble metal NP, polymer beads, and lanthanide-based NP [127, 174–177]. ICP-MS has been frequently used to quantify FG-bearing ligands on gold NP [169, 173, 178–180], but has also been applied to quantify FG and ligands on other NM such as silica NP, polymeric beads, and semiconductor QD [126, 166–168]. Particularly single-particle ICP-MS (sp-ICP-MS) is a promising technique as in principle it allows to simultaneously measure the particle number concentration and the number of FG/ligands on the surface of individual particles. However, especially for light elements often present in commonly used organic FG and ligands, that are difficult to ionize (large ionization potential) and are prone to a high background, quantification is very challenging and detections limits are higher compared to other heavier elements. Further instrument improvements and methodological advances can make sp-ICP-MS a very well-suited method for NM characterization [181–183].

In principle, atomic absorption spectroscopy (AAS) with either flame-based or electrothermal (graphite tube) atomizers can also be used to quantify FG and ligands, but as AAS is mostly limited to metallic and semi-metallic elements that are typically not present in organic ligands and surface groups, this technique is of very limited use for FG analysis [184]. However, there exists a special AAS technique that is very sensitive to fluorine [185], which can be utilized for the quantification of FG derivatized with fluorine-containing labels, such as BODIPY dyes for the validation of optical assays or other elemental tags bearing CF_3_ groups for the comparison with XPS measurements or ^19^F-NMR (see Fig. 8).

Other MS methods that can also be used for NM surface characterization, but are not further detailed here, are time-of-flight secondary ion mass spectrometry (ToF-SIMS) and matrix-assisted laser desorption/ionization (MALDI) in combination with time-of-flight (ToF) analysis of the released ions by mass spectrometry (MALDI-ToF-MS). These methods can provide molecular information of FG and surface bound molecules [186–188], but up to now, have been rarely utilized for NP analysis.

### Vibrational spectroscopy

Vibrational spectroscopy measures the absorption or (inelastic) scattering of incident light due to vibrational stretching and/or bending modes of molecules within a sample. Infrared (IR) spectroscopy relies on the absorption of IR radiation, typically in a wavelength region between 2500 nm and 25 μm (4000–400 cm^1^ on the wavenumber scale). The fundamental vibrations of most chemical bonds occur within this spectral region. IR spectroscopy is frequently being used for qualitative and quantitative analysis of organic compounds in agriculture, food products, polymers, pharmaceuticals, cosmetics and the petroleum industry as well as for the monitoring of chemical reactions in process analysis [189–191]. Nowadays, commonly Fourier-transform infrared (FTIR) spectroscopy is used, allowing for the simultaneous detection of all vibrational frequencies [192, 193]. Raman spectroscopy is a complimentary vibrational spectroscopic technique, which detects inelastically scattered photons (Raman scattering) from a monochromatic light source (usually a laser) in the near-UV to near-IR range. Due to the different selection rules valid for both methods, Raman spectroscopy yields similar—yet complementary—information compared to IR spectroscopy [194]. Both methods are relatively fast and non-destructive, and can be coupled with other analysis methods. For FG and ligand analysis on NM, surface-sensitive variants of these techniques are of particular importance like attenuated total reflection (ATR-) FTIR [195, 196], diffuse reflectance infrared Fourier transform spectroscopy (DRIFTS) [197, 198], and surface-enhanced Raman spectroscopy (SERS) [199–201]. While SERS is limited to electrically conducting materials such as graphene or noble metals, FTIR spectroscopy is frequently employed to study a wide range of NM. Both DRIFTS and ATR-FTIR overcome the shortcomings of sample preparation complexity in classical FTIR spectroscopy, and ATR-FTIR further allows in situ characterization of particle surfaces in biologically and environmentally relevant media [196]. Although vibrational spectroscopy is widely accepted for qualitative analysis of NM surfaces [202–205], there are only few literature reports on the application of these methods for quantitative analysis. Nevertheless, with modern instrumentation and combined approaches, the quantitative determination of FG and ligands at the nano- to picogram level should be feasible [206, 207]. Examples for FG determination with ATR-FTIR spectroscopy include the measurement of the density of thiol and bromoalkyl FG on silica particles [208] and the amount of APTES on silica-coated iron oxide NP [209]. Furthermore, ATR-FTIR can be applied to characterize and (semi-)quantify the chemical composition of mixed ligand layers at NM surfaces [210, 211]. To quantify FG and surface ligands with FTIR spectroscopy, a calibration curve is generally required and samples that obey the Beer-Lambert law. A combined use of chemometric tools such as convolutional neural networks and ensemble learning, with different vibrational spectroscopy techniques, might contribute to an increased accuracy of quantitative FG analysis in the future [212].

### X-ray-based methods

X-ray photoelectron spectroscopy (XPS) measures the number and kinetic energy of electrons that escape from the near-surface region of a sample, like a planar substrate or particles deposited on a substrate, up to an information depth of 5–10 nm upon irradiation with an X-ray beam in vacuum. In laboratories, typically Mg Kα or Al Kα sources with photon energies of 1253.6 eV or 1486.6 eV are used. The information depth of XPS is determined by the inelastic mean free path (IMFP) of the photo-excited electrons in solid matter. Using higher photon energies between 3 and 15 keV as in hard X-ray photoelectron spectroscopy (HAXPES) can extend the information depth up to about 100 nm [213]. XPS measurements provide information on surface composition, the enrichment or depletion of elements at the surface, the presence and/or thickness of coatings, and the chemical states of the elements [23, 214, 215]. Therefore, information on the chemical composition and amount of FG or certain chemical species at the particle surface can be obtained [216]. Due to the sensitivity of XPS for all elements except H and He, not only inorganic surface coatings, passivation shells, and surface modifications by chemical processes like oxidation or etching can be determined [217–219], but also organic coatings, ligands, and surface-bound biomolecules [220, 221]. Depending on the chemical composition of the sample and the surface ligands or FG, XPS can be done without the need for a label. A reliable quantification requires calibration with suitable reference material or standards [222]. Alternatively, theoretically derived values for the cross sections (Scofield factors), the IMFP and the transmission function of the spectrometer can be used [214, 223–225]. It must be noted that all these quantification procedures are only valid to homogeneous planar surfaces; however, there are different approaches which can be used for NP [214]. To enhance the sensitivity and selectivity of XPS and/or to enable the comparison with other more quantitative methods like NMR for method calibration and validation, for example fluorine-containing reactive labels like 3,5-bis(trifluoromethyl)phenyl isothiocyanate [226], trifluoroacetic anhydride (TFAA) [227], and 2,2,2-trifluoroethylamine (TFEA) [158] can be employed for the chemical derivatization of specific surface FG like amino, hydroxyl, and carboxy FG, respectively. The use of TFEA to quantify the amount of carboxy groups on PMMA particles with a grafted shell of PAA also provides a link to the SI unit mole, as shown in Fig. 8 [157, 158].

In the last years, an increasing number of examples for the applicability of XPS to determine the elemental distribution of small inorganic NM and the chemical surface modification of various NP by light-induced chemical changes, ligand exchange, or the presence of certain species like chloride ions has been reported [23, 28, 219, 228]. XPS was also used to derive the thickness of inorganic passivation shells, e.g., on semiconductor QD like CdSe/CdS by simulation of the spectra [214]. As the size of many NM is larger than the XPS information depth, and as NP size, shape, and morphology determine the fraction of surface elements accessible within this information depth, the use of XPS for the quantification of NM ligand shells and FG is challenging and requires mathematical modeling of the measured data, e.g., with a software like SESSA (Simulation of Electron Spectra for Surface Analysis) [229], thereby also considering the size- and shape-dependent curvature of the NM surface. New approaches for the quantification of XPS at nanostructured materials take these effects into account, but further developments are necessary for more complex materials [214, 230–233]. Another challenge of XPS measurements is the need of an appropriate sample preparation and handling to prevent changes of the NM due to undesired influences from the surrounding of the particles [23]. Also, NM agglomeration and aggregation as well as decomposition or changes induced by the X-ray beam can influence the reliability of the obtained results.

Another X-ray based analytical technique that is in principle capable of quantitatively measuring the elemental composition of a sample presents X-ray fluorescence (XRF) spectroscopy, which detects the secondary (“fluorescent”) X-rays emitted from a material after excitation with high-energy X-rays (or sometimes gamma rays) using either energy-dispersive (EDXRF) or wavelength-dispersive (WDXRF) spectroscopy [234]. As the secondary radiation from lighter elements (with Z < 12) is of relatively low energy (< 3 keV) and has a low penetration depth, it is often reabsorbed by the sample and severely attenuated by any matter between the sample and the detector. Hence, XRF is usually only applied to characterize the chemical composition or impurities on inorganic materials such as metal, glass, ceramic, and semiconductor surfaces, films, or layers. However, using vacuum technique, synchrotron radiation, and/or special detector windows also lighter elements such as carbon, nitrogen, oxygen, and fluorine can be detected [234–236]. Particularly the surface-sensitive methods total reflection X-ray fluorescence (TXRF) and grazing-incidence X-ray fluorescence (GIXRF) spectroscopy using X-ray standing waves (XSW) are principally well suited for a reference-free quantification of FG on NM [237–239], but for particle samples additional corrections are necessary to account for absorption and shadowing effects occurring on the nanostructured surfaces [240].

### Other techniques to determine the total number of FG

Other techniques that can be applied for FG and ligand characterization and are more or less well suited for a quantitative analysis include elemental analysis (EA), thermal analysis methods such as thermogravimetric analysis (TGA) and differential scanning calorimetry (DSC), and separation techniques like asymmetrical flow field-flow fractionation (AF4).

EA is an inexpensive method to determine the elemental composition of a material by combusting the sample under controlled conditions and analyzing the combustion products quantitatively. Particularly the so-called CHNS analysis, that provides the mass fractions of carbon, hydrogen, nitrogen, and sulfur via their combustion gases under high temperature and high oxygen conditions (converting these elements to their oxidized form, i.e., to CO_2_, H_2_O, NO or NO_2_, and SO_2_, or reducing, e.g., NO or NO_2_ to N_2_), is in principle well suited for FG or ligand quantification on NM, as long as the NM matrix does not contain the FG-specific element(s). These gases are then detected by a thermal conductivity detector that typically is calibrated daily with suitable standards or reference materials. However, CHNS analysis has only rarely been used for FG quantification, e.g., for carboxy groups on alumina particles, [241] or amino groups on graphene oxide [242] as well as silica particles [243, 244], due to relatively large amounts of sample needed and the relatively low detection sensitivity compared to other methods like ICP-MS [126]. For NM characterization, CHNS analysis is often used only in conjunction with other analytical methods such as FTIR, TGA, or XPS [126, 242, 243, 245].

TGA and DSC are typically used to obtain information on the thermal stability, chemical composition, and purity of a sample as well as on kinetic parameters. DSC can also be employed to determine phase transitions, e.g., of liquid crystals. TGA detects the resulting mass changes as a function of temperature under defined conditions, while DSC measures the difference in the amount of heat required to increase the temperature of the sample [246]. Recent results of the EU funded project *ProSafe* identified TGA as a very useful, simple, and reliable method to study the surface chemistry particularly of inorganic NM like silica, metal, and lanthanide-based NP as well as QD [247]. For TGA measurements, no complex sample preparation is needed. However, due to the underlying measurement principle, all contaminants present in the sample such as remaining dispersion media/solvents, impurities originating from NM synthesis, and free ligands/molecules can contribute to the mass changes, and thus, influence the results. Therefore, a careful work-up and clean-up procedure of the NM sample to be analyzed is mandatory. Also control samples and precise heating steps at lower temperatures are often included to overcome the inherent challenge of undesired mass contributions from matters other than the organic surface ligand/FG [164, 248]. Another drawback is the amount of NM needed for a single TGA analysis, i.e., commonly several milligrams, which can make TGA less suitable for small-scale samples of functionalized NM used in biomedical applications. Modern TGA methods address these limitations by a higher sensitivity. Mansfield et al. [246] developed a microscale TGA method (μ-TGA) using a quartz crystal microbalance, that needs a 1000-fold reduced amount of sample. With this method, the authors could obtain results for commercial CNT, polymer-coated gold NP as well as polymer-modified gold/silica NP which were comparable with those determined by conventional TGA [246, 249, 250]. They also used μ-TGA to quantify the amount of surface-bound poly-l-lysine and DNA on gold NP intended for potential use in biomedical applications [250]. Another advanced TGA approach presents the coupling with mass spectrometry (TGA-MS) or FTIR spectroscopy (TGA-FTIR) to enable the identification of the species responsible for the observed mass loss upon heating. For example, TGA-MS has been used to determine the amount of aromatic molecules adsorbed on CNT [251] and to identify the organic compounds extractable from various NM [252]. Moreover, TGA has been used in multi-method approaches for quantitative analysis to enhance the reliability of the results by comparing them with information from FTIR, NMR, ICP-MS/OES, and XPS measurements [164, 175, 186, 253–256]. And just recently, TGA has been applied to quantify adsorbed citrate molecules on the surface of gold NP, which provided new insights into mechanistic details of the well-known *Turkevich* gold NP synthesis method [257].

As the density of FG/ligands on the NM surface determines its charge and hydrophilicity/hydrophobicity, also NP separation techniques like AF4, a fractionation method that is used for the characterization of polymers, proteins, and NP, coupled with capillary electrophoresis (CE) can be used for FG quantification. Combining AF4 and CE provides separation by size and surface charge. The correlation of these parameters requires a calibration curve with similarly sized particles of known FG density to correlate the CE retention times with FG density [258]. This method is, however, only suited for surface FG that control particle charge, e.g., for (de)protonable functionalities such as carboxylic acids and amines. Moreover, in addition to the method-inherent limitation of AF4, this method combination faces the same limitations as electrochemical titration methods, except for signal contributions from the presence of other (de)protonable molecules due to the coupling with a NP separation technique.

## Conclusion and future challenges

All analytical techniques presented here for the analysis and quantification of functional groups (FG) and ligands on nanomaterial (NM) surfaces possess specific method- and material-related requirements and limitations. For a straightforward, efficient, and reliable quantification of FG and ligands on NM, these parameters need to be considered. Also, the distribution of NM size, shape, and surface morphology must be taken into account for a thorough determination of the FG/ligand density (number of FG/ligands per particle or surface area), which can vary from particle to particle in a NM population. In addition, it must be kept in mind whether the total number or the derivatizable number of FG is desired for which purpose/application and with which uncertainty. For example, quality control during NM fabrication and surface modification or the bioconjugation of NM reporters to bioligands for fluorescence assays do not necessarily require the knowledge of the total number of FG. For the production and characterization of nanoscale reference materials, e.g. as negative and/or positive controls in toxicity tests or for the application in the health sector or as food additives can impose more stringent requirements on NM characterization. To provide guidelines for the choice of the optimum method(s), an overview of the most relevant criteria for the choice of suitable methods for FG and ligand quantification on NM is summarized in Table 1.

**Table 1:** Overview of the analytical methods for FG and ligand quantification on NM covered by this review, classified according to their signal generation principle and utilized reporters, with typical examples and possible FG that can be targeted as well as the type of FG measured (total FG or derivatizable FG). Important parameters such as the necessity of a label, sample requirements, and the need for method calibration are also summarized

Analytical method		Description/reporter (Examples)	Functional groups/Ligands		Requirements			Ref.
			Target	Type	Label	Sample	Calibration	
Electrochemical titration	Potentiometric titration	Electrochemical potential as function of added H^+^/OH^−^	All (de)protonable FG (e.g., -COOH, -NH_2_, -SH)	Total FG	Label-free	Aqueous dispersion, free of (de)protonable contaminants	Calibration with titrants of known concentration	[75–88]
	Conductometric titration	Conductivity as function of added H^+^/OH^−^	All (de)protonable FG (e.g., -COOH, -NH_2_, -SO_4_)					[63, 64, 89–100]
	Boehm titration	Potent. titration with bases of different p*K*_a_, back titration of residual titrant	Oxygen containing, acidic FG					[101–113]
Optical spectroscopy	Labeling with conventional dyes	FITC, Dansyl chloride	Corresponds to reactive group of the label (e.g., -NH_2_, -COOH, -N_3_)	Derivatizable FG	Dye labeling	Aqueous dispersion, free of contaminants bearing the same FG	Calibration required: Knowledge of the reporter’s optical properties used for quantification (ε_λ_ and/or Φ_PL_) needed	[63, 64, 114, 117–119]
	Activatable or chromogenic dyes	Fluram, Ninhydrin, Ellman’s reagent	Corresponds to reactive group of the dye (e.g., -NH_2_, -SH)		Dye formation upon reaction			[64, 65, 114, 115, 120–129]
	Cleavable reporter	Fmoc, SPDP, 4-NBA	Corresponds to reactive group of the probe (e.g., -NH_2_, -COOH, -N_3_)		Labeling and release of dye reporter			[64, 89, 123, 124, 130–142]
	Adsorptive reporter	Toluidine Blue, Ni^2+^	All charged FG (e.g., -COO^−^, -NH_3_^+^)	Total FG or derivatizable FG^a^	Adsorption/ desorption equilibrium	Aqueous dispersion		[63, 116, 143–145]
Other analytical techniques	NMR	Incl. quantitative solid-state NMR	FG containing elements with intrinsic magnetic moment (^1^H, ^13^C, ^19^F, ^31^P)	Total FG or derivatizable FG^b^	Label-free or reporter-based^b^	Dispersion in deuterated solvent, or solid state	Calibration required with internal standard of known purity	[29, 51, 63, 89, 121, 123, 146–164]
	ICP-MS/OES/AAS	Incl. coupled techniques and single particle ICP-MS	Most elements, but not H, C, N, O; is often used to determine NM conc.		Label-free or reporter-based^b^	Any physical state	Calibration required	[64, 89, 126, 127, 165–188]
	FTIR/Raman	Incl. ATR-FTIR, DRIFTS, and SERS	All FG with IR/Raman-active transition bands		Label-free or reporter-based^b^	Any physical state, free of contaminants	Calibration or chemometric tools required	[189–212]
	XPS/XRF	Incl. HAXPES and TXRF/GIXRF	All elements except H, He, Li		Label-free or reporter-based^b^	Sample deposited on planar substrate	Calibration required	[23, 28, 157, 158, 213–240]
	TGA/DSC	Thermal analysis (mass change or heat quantity)	In principle all organic FG/ligands	Total FG	Label-free	Powder, dispersion, film (TGA), or gel (DSC); free of contaminants	Calibration-free	[164, 175, 186, 246–257]
	EA	Quantitatively analysis of combustion products	C/H/N/S-containing FG, O and F also possible			Any physical state	Calibration required	[126, 241–245]

^a^Depending on the size of the adsorptive reporter: small metal ion reporters will yield the total number of FG (or a value very close to this number), while larger dye reporters will yield a lower number of derivatizable FG

^b^Depending on the chemical nature of the NM and the FG of interest, either intrinsically present moieties (label-free) or specific reporters (label-based methods) can be utilized for signal generation and quantification, yielding either the total or the derivatizable number of FG

Electrochemical titration methods are inexpensive, rapid, require only simple and widely available laboratory instrumentation, and can be performed by technical staff under ambient conditions at constant temperature. Also, data interpretation is comparatively easy and straightforward. This makes these methods well suited for routine analysis as well as production and quality control of synthesized or functionalized NM. Sometimes the removal of CO_2_ from air is mandatory by purging sample solutions and dispersions with nitrogen or argon. Moreover, it must be assured that complete (de)protonation has been reached after each titration step. All electrochemical methods provide the total amount of the FG of interest present in the sample, i.e., on the NM surface, as the reporters used for signal generation, namely protons or hydroxide ions, are very small [64, 89]. This has been verified, e.g., by comparing the results from conductometry and quantitative solid state NMR measurements [63]. In principle, also the number of derivatizable FG can be obtained via electrochemical measurements by comparing the results prior to and after FG derivatization, combining electrochemical titrations with other quantification methods such as optical spectroscopy utilizing dye reporters. A general drawback of electrochemical methods is their lack of specificity and selectivity, as they measure all (de)protonable species with comparable p*K*_a_ values present in the sample, and hence, not necessarily only the number of FG on the NM surface. As previously mentioned, other species remaining from NM synthesis like surfactants, initiators, stabilizers, or excess ligands with (de)protonable groups can also contribute to the measured signals, and thus, can distort the obtained results if not removed prior to analysis. Also, other (de)protonable FG on NM can interfere with the signals originating from the target. For example, for NM bearing a mixture of different types of FG like carboxy and amino groups, the simultaneous quantification of the total amount of both types of FG is very challenging if not impossible [64]. This implies also, that certain particle matrices can interfere with electrochemical titrations like mesoporous silica where the close match of the p*K*_a_ values of the silanol groups (p*K*_a_ 4.5–5.5) and carboxy groups p*K*_a_ (about 4.8) prevents a discrimination between these FG. Moreover, the NM needs to be colloidally stable during the course of the acid-base titration, i.e., at the pH conditions necessary for (de)protonation of the FG/ligand of interest. For example, the dissolution of the silica matrix at alkaline pH values required for the determination of amino groups (p*K*_a_ about 9–11) renders the electrochemical quantification of amino FG on silica not feasible [89]. In addition, electrochemical titration methods like conductometry require a relatively large amount of sample (about 10–20 mg/mL of the surface functionalized NM), which renders these methods suitable only for relatively simple and self-made particles that are either not expensive or can be easily prepared in larger quantities. For expensive NM that are difficult to obtain on a large scale or for surface ligands which are either costly or difficult to synthesize, other methods that require less amount of sample present a better choice like optical assays.

Optical assays, particularly fluorometric assays, are very sensitive, require only small amounts of sample, and can be performed by technical staff with standard bench-top instrumentation available in most laboratories. This makes them ideal for routine process monitoring during particle manufacturing and for quality control of surface functionalization. Moreover, optical measurements are simple and fast, and the chemical derivatization step necessary for labeling-based assays can provide an additional selectivity. Meanwhile, there is a large toolbox of differently sized optical reporters with various reactive groups commercially available including the different types of reporters introduced in the section on optical assays. However, for all optical assays as well as for other analytical methods relying on labeling reactions, only the number of derivatizable FG is obtained and the result can be affected by a combination of the reactivity of the label or reporter, the underlying reaction mechanism, the reaction yield, and particularly for crowded surfaces, also by reporter size, shape, and charge. Depending on the NM application, the influence of reporter size and shape must not be a disadvantage but can provide a more realistic estimate of the number of FG that can be derivatized with the molecule of interest. For bioconjugation reactions involving large biomolecules, commonly, the number of derivatizable FG determined with a reporter such as an organic dye provides an upper limit of the FG on the NM surface that can be coupled to the respective biomolecule. A general drawback of optical methods is the need for a suitable calibration to correlate the intensity of the measured optical signal to the analyte concentration, i.e., the amount of FG or ligands. The calibration needs to consider the sensitivity of the reporter’s optical properties to its microenvironment, which can change the spectroscopic properties relevant for quantification, i.e., the reporter’s molar absorption coefficient and especially its QY_PL_ values. This can be accomplished by choosing a standard for assay calibration that closely matches the reporter and its environment in the optical assay. This can make an accurate calibration (i.e., measurement uncertainties < 5%) tedious and can render the calibration sample specific. However, if larger uncertainties exceeding 20% are acceptable, a universal calibration could be sufficient. Also, optical signals can be distorted by interferences from the sample material like size- and wavelength-dependent light scattering as well as NM absorption and/or emission. For fluorescent labels that are detected when bound to the NM surface, quantification can also be hampered by reporter-specific and labeling density-dependent dye-dye interactions resulting in fluorescence quenching. Such sources of uncertainty can be elegantly circumvented with the aid of cleavable probes and catch-and-release assays.

Analytical techniques like NMR spectroscopy, ICP-MS, and XPS are very valuable tools for FG quantification and can yield the number of total and/or derivatizable FG depending on the respective surface-modified NM and the reporter used for signal generation. As detailed before, utilizing intrinsically present moieties provides the total amount of a certain FG, while the use of specific reporters in conjunction with chemical derivatization/labeling reactions yields the number of derivatizable FG. Moreover, particularly NMR and XPS can simultaneously provide information on mixed ligand shells and different FG. Drawbacks of these methods are, however, the need for expensive and sophisticated instrumentation, well-trained scientific staff, and elaborated data analysis. Therefore, such methods are often not the optimum choice for routine analysis and quality control; here, simple electrochemical methods and optical assays are better suited. However, these analytical methods are essential for validating the results obtained with simpler methods. Elegant tools for method validation present multimodal reporters that can be read out with different analytical techniques varying in the principle of signal generation, as summarized in the previous sections. Also, methods like NMR are mandatory to establish a traceability chain to the SI unit mole.

In the future, the increasingly recognized importance of FG and ligand quantification on NM and its direct correlation with the safe(r) use of NM will require more interlaboratory comparisons using different analytical methods to determine accomplishable uncertainties for broadly used NM and typical surface modifications. This is particularly relevant for applications of NM in the life sciences, health sector, and as food additives, and the corresponding quality control of NM production and long-term stability. Due to the application-specific importance of information on the total and derivatizable number of FG/ligands, analytical methods for both types of surface functionalities are needed. For the latter, the information obtained is influenced by the size, shape, and charge of the applied reporter, also in comparison to the respective properties of the molecule(s) of interest that are to be attached to the FG/ligands on the NM surface. Thus, models are desired that enable to consider these features. A relatively simple approach would be to calculate or estimate the steric demand (footprint) of the reporter label and the molecule of interest, which, however, needs to be verified at least for a set of commonly used reporters and application-relevant target (bio)molecules such as typical peptides, oligonucleotides, and proteins including antibodies and enzymes. In any case, the overall aim should be to establish protocols for surface FG/ligand analysis and quantification (with known uncertainties) for commonly employed methods, and to eventually standardize these methods. Such activities are currently being pushed forward, e.g., by ISO/TC 229 *Nanotechnologies*, the Nanomaterials Study Group of ISO TC201 SG1, and ISO TC 201 *Surface Chemical Analysis* as well as different working groups of IEC. Other activities are being pursued by national standardization organizations, e.g., by the XPS community. Particularly attractive and efficient tools for surface group analysis are multimodal reporters, which enable to correlate the results obtained with different analytical methods, thereby simplifying method comparison, validation, and traceability, as exemplarily shown in Fig. 8.

Protocols and recommended methods for surface analysis are also needed for establishing nanoscale reference materials for method calibration and/or validation, as reliable control for toxicity tests, and the supply of reference data of NM. Other properties of NM, that are closely linked to surface chemistry and will be of increasing importance for nanotechnology and nanobiotechnology in the future, are NM hydrophobicity/hydrophilicity, NM stability (including the possible release of potentially toxic constituents), and environment-induced changes in NM surface chemistry (including adsorption of (bio)molecules, (bio)corona formation, etc.) for representative and application-relevant test scenarios. Here, also overall accepted methods are needed. Although these needs have been meanwhile recognized by metrological institutes, standardization organizations, and regulatory agencies worldwide, the constantly increasing number of new and more advanced NM developed make it difficult to keep track. A categorization or classification of NM could present an appropriate tool that has been addressed by different EU consortia, and stronger requirements on the quality of the analytical data to be provided for scientific publications involving NM could be beneficial to improve the overall confidence in “nano” data [31, 32, 41, 247, 259].

## Data Availability

Not applicable.
